# High-throughput analysis of the transcriptional patterns of sexual genes in malaria

**DOI:** 10.1186/s13071-022-05624-w

**Published:** 2023-01-13

**Authors:** Abel Cruz Camacho, Edo Kiper, Sonia Oren, Nir Zaharoni, Netta Nir, Noam Soffer, Yael Noy, Bar Ben David, Anna Rivkin, Ron Rotkopf, Dan Michael, Teresa G. Carvalho, Neta Regev-Rudzki

**Affiliations:** 1 Faculty of Biochemistry, Department of Biomolecular Sciences, Weizmann Institute of Science, 7610001 Rehovot, Israel; 2 Department of Life Sciences Core Facilities, Weizmann Institute of Science, 7610001 Rehovot, Israel; 3 Feinberg Graduate School, Weizmann Institute of Science, 7610001 Rehovot, Israel; 4 Department of Microbiology, Anatomy, Physiology and Pharmacology, La Trobe University, Melbourne, VIC 3086 Australia

**Keywords:** Malaria, Gametocytogenesis, RT-qPCR, Gene expression, *Plasmodium falciparum*, Gametocyte, Automatization

## Abstract

**Background:**

*Plasmodium falciparum* (*Pf*) is the leading protozoan causing malaria, the most devastating parasitic disease. To ensure transmission, a small subset of *Pf* parasites differentiate into the sexual forms (gametocytes). Since the abundance of these essential parasitic forms is extremely low within the human host, little is currently known about the molecular regulation of their sexual differentiation, highlighting the need to develop tools to investigate *Pf* gene expression during this fundamental mechanism.

**Methods:**

We developed a high-throughput quantitative Reverse-Transcription PCR (RT-qPCR) platform to robustly monitor *Pf* transcriptional patterns, in particular, systematically profiling the transcriptional pattern of a large panel of gametocyte-related genes (GRG). Initially, we evaluated the technical performance of the systematic RT-qPCR platform to ensure it complies with the accepted quality standards for: (i) RNA extraction, (ii) cDNA synthesis and (iii) evaluation of gene expression through RT-qPCR. We then used this approach to monitor alterations in gene expression of a panel of GRG upon treatment with gametocytogenesis regulators.

**Results:**

We thoroughly elucidated GRG expression profiles under treatment with the antimalarial drug dihydroartemisinin (DHA) or the metabolite choline over the course of a *Pf* blood cycle (48 h). We demonstrate that both significantly alter the expression pattern of *PfAP2-G*, the gametocytogenesis master regulator*.* However, they also markedly modify the developmental rate of the parasites and thus might bias the mRNA expression. Additionally, we screened the effect of the metabolites lactate and kynurenic acid, abundant in severe malaria, as potential regulators of gametocytogenesis.

**Conclusions:**

Our data demonstrate that the high-throughput RT-qPCR method enables studying the immediate transcriptional response initiating gametocytogenesis of the parasites from a very low volume of malaria-infected RBC samples. The obtained data expand the current knowledge of the initial alterations in mRNA profiles of GRG upon treatment with reported regulators. In addition, using this method emphasizes that asexual parasite stage composition is a crucial element that must be considered when interpreting changes in GRG expression by RT-qPCR, specifically when screening for novel compounds that could regulate *Pf* sexual differentiation.

**Graphical Abstract:**

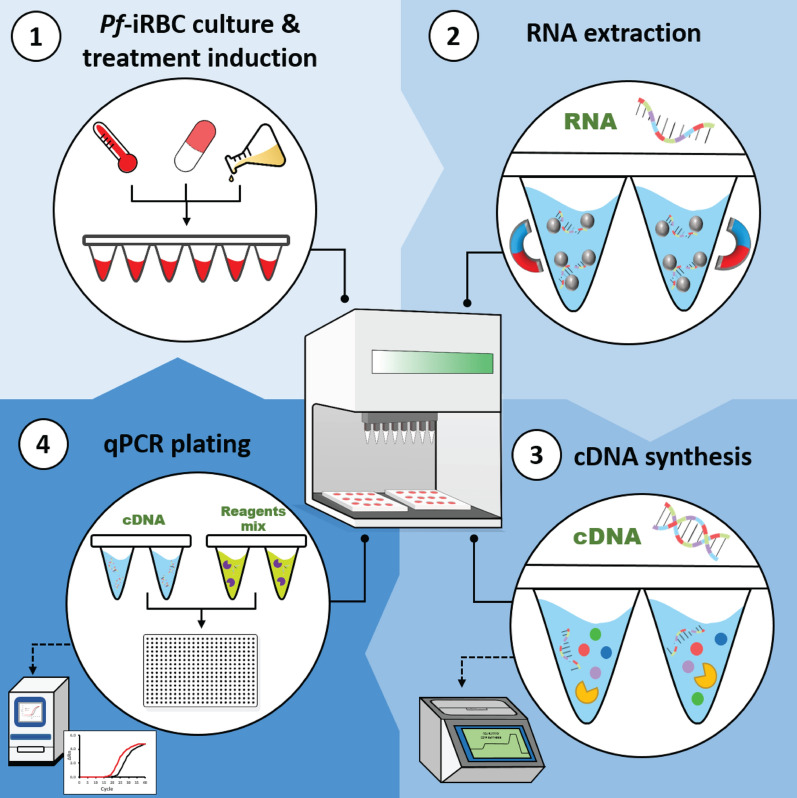

**Supplementary Information:**

The online version contains supplementary material available at 10.1186/s13071-022-05624-w.

## Background

Malaria, a parasitic disease caused by species of the apicomplexan genus *Plasmodium*, continues to be a major human health problem, affecting millions of individuals and causing more than 600,000 deaths in 2022 alone [1]. *Plasmodium falciparum* (*Pf*) is the most virulent species, representing > 99.7% of the malaria cases in Africa [2]. *Plasmodium* parasites have a complex life cycle that includes two hosts, the human and the *Anopheles* mosquito vector [3]. The mosquito vector injects infective sporozoites into the human host during its blood feed. After an initial replication cycle in the liver, the parasites reach the blood vessels and invade red blood cells (RBCs) to establish the blood-stage infection consisting of numerous 48-h cycles of parasitic asexual multiplication [3]. During the asexual blood cycles, *Pf* invades the host RBC and develops into the ring stage, grows by consuming the host hemoglobin as a trophozoite, divides its genetic material numerous times during the schizont stage and gives rise to a new generation of infective merozoites that renew the erythrocytic cycle [4, 5].

A small subset of the asexual parasites (< ~ 5%) [6] undergoes a sexual differentiation process known as gametocytogenesis, during which the parasites transform into mature female or male gametocytes within 10–12 days [7]. Mature gametocytes represent an essential stage in the parasite’s life cycle, as they are taken up and develop inside the mosquito vector, thus being responsible for the transmission of malaria [8]. Gametocytogenesis serves therefore as an extremely attractive drug target process to halt malaria transmission [9].

The molecular mechanisms that regulate sexual commitment, as well as the cascade of events within the pathway, remain mostly elusive [6, 10]; however, a key parasitic regulator, *PfAP2-G*, has been recently identified [11, 12]. AP2-G is a transcriptional factor with a single AP2 domain that binds to the GNGTAC DNA motif in the promoters of many gametocyte-related genes (GRGs) [12] and induces their expression [13]. Some of these GRGs include previously known early gametocyte markers, such as *Pfs16, Pfg27* [14–16]*, Pfg14.744* and *Pfg14.748* [17] as well as sexual ring stage markers such as *gexp05* [18] and *gexp02* [13, 19]. A ChIP-seq study revealed that *PfAP2-G* directly binds to the promoters of all of these genes and induces their expression [13].

In most parasites, the sexual commitment decision takes place at least one erythrocytic cycle before the actual differentiation process [5, 10, 20]; however, under certain induction circumstances, parasites can directly differentiate into the sexual stages in the same induction cycle [20, 21]. *PfAP2-G* is epigenetically silenced in asexual parasites by the repressive histone mark H3K9me3 as well as the action of the heterochromatin protein 1 (PfHP1) and the histone deacetylase 2 (Hda2) [22–26]. Under the influence of partially known signals, upstream regulating proteins such as *Pfgdv1* become activated and remove the repressive epigenetic marks on the *PfAP2-G* locus [27, 28], activating *PfAP2-G* in specific asexual parasites, generating sexually committed schizonts. The merozoites that emerge from the committed schizonts invade naïve RBCs, causing them to generate sexual rings; these then undergo a *PfAP2-G-*dependent program and eventually differentiate into gametocytes [9, 10]. An inducible system revealed that *PfAP2-G* is mostly expressed during the late schizont and ring stages, and its expression declines in the trophozoites during the sexual commitment cycle [21]. The timing of *PfAP2-G* stabilization determines if the parasites will differentiate into gametocytes immediately or after one cycle as sexually committed parasites [21].

Despite the substantial efforts to dissect the molecular pathway underlying gametocytogenesis, intrinsic and extrinsic molecular regulators remain mostly unknown [9]. The current hypothesis is that gametocytes are steadily produced at each asexual cycle due to the stochastic activation of *PfAP2-G* among a subset of the parasitic population [10, 12]. In other words, it is believed that a basal gametocytogenesis commitment rate (GCR) exists and that environmental, parasitic- or host-derived factors play a role in altering the basal GCR [9, 12].

Host-derived factors such as white blood cell count, host mean temperature [29, 30], hematocrit [31], age [31] and reticulocyte abundance [32] have been previously correlated with changes in the levels of parasitemia and GCR; however, none of these factors have shown consistent transcriptional induction of gametocytogenesis [6]. Importantly, lysophosphatidylcholine (LysoPC), a host-derived phospholipid present in human serum, has been shown to act as a repressor of gametocytogenesis [33]. LysoPC participates in the Kennedy metabolic pathway to synthetize phosphatidylcholine (PC), a central membrane phospholipid in malaria [34]. Depletion of LysoPC or its downstream metabolites choline and PC, as well as restriction of the Kennedy pathway, has been shown to induce gametocytogenesis in vitro *Pf* cultures [28, 33, 35, 36].

Stress-inducing environmental conditions are also linked to sexual conversion [37–40], as they may activate the parasite to develop an “escape strategy” in response to the host immune response [4, 7] or to antimalarial drugs [38]. Chloroquine, an antimalarial drug, was associated with higher gametocytemia [41], as were other antimalarial compounds such as atovaquone, methylene blue [42] and artemisinin [35]. Nevertheless, a recent study demonstrated that changes in GCR due to drug stress may be part of a general stress response rather than triggered by compound-specific mechanisms [40]. Metabolites such as lactic acid and homocysteine, which are upregulated during metabolic acidosis in severe malaria and in oxidative stress, respectively, are thought to activate sexual conversion [43, 44]. Additionally, kynurenic acid is another metabolite highly abundant in cerebral malaria patients [45, 46], and although its role in gametocytogenesis remains unclear, it was previously reported to aid in gametogenesis in the mosquito gut [47]. Collectively, these data suggest that a stress response is involved in *Pf* sexual conversion.

Gametocytogenesis is the single most critical process for malaria transmission; however, current laboratory methods for studying this process are lengthy and tedious, limited in throughput and mostly based on direct quantification of sexual parasites. Gametocytogenesis has been classically assessed in vitro by microscopic quantification of gametocytemia in Giemsa-stained blood films [48, 49], which requires culturing the parasites over the course of at least 4–7 days to allow for the development of gametocytes [48–50]. The main pitfall of this lengthy procedure is the requirement for daily culture maintenance [49]; hence, it is not suitable for high-throughput screenings.

Transgenic parasite lines expressing fluorescent reporters fused to the promoters of gametocyte-specific genes are widely used to estimate the sexual commitment level [17, 42, 51, 52]. The use of transgenic lines to estimate the percentage of GCR (%GCR) enables higher-throughput screening of potential candidate treatments by means of flow cytometry [19]. However, some of these systems present technical challenges, such as the loss of expression of episomal transgenes and background expression in asexual stages [19]. Transgenic lines with stable CRISPR-mediated transfection proved to be powerful and accurate tools for GCR estimation [19, 35]; yet, they remain dependent on gene expression analysis, as the transgene expression may not necessarily reflect changes in expression of the endogenous gametocyte genes.

Gene expression techniques such as RT-qPCR have not been widely used for studying early gametocytogenesis, despite their common use in diagnostic assays for molecular detection of parasites in patients [53, 54]. Overall, no high-throughput platforms for assessing the immediate changes in sexual conversion by monitoring RNA expression have been developed to date.

Here, we developed a robot-automated RT-qPCR platform to robustly evaluate changes in the mRNA expression profile of *Pf.* Particularly, we concentrated on profiling the expression of known sexual marker genes in up to hundreds of samples simultaneously at high resolution. Since gametocytogenesis is essential for malaria transmission, in this study we focused on the early transcriptional regulation of gametocytogenesis, as an example of the plethora of processes in *Pf* biology where transcriptional regulation of gene expression is crucial, to include for example monitoring virulence *var* gene expression [55], invasion gene expression [56], sex determination and stage transition [57].

Our advanced multi-step technology consists of three hands-free steps: RNA extraction, cDNA synthesis and RT-qPCR reaction plating. Adopting our detailed systematic method could advance the study of triggers involved in this crucial stage of malaria transmission by elucidating mechanistic insights on transcriptional processes during early gametocytogenesis. We therefore designed our platform to simultaneously monitor up to 10 different gametocyte-related genes (GRG) from a large scale of samples using very low volumes of *Pf*-infected RBCs (*Pf*-iRBCs).

Furthermore, using our advanced platform, we evaluated the effects of both choline, a previously studied regulator of *Pf* sexual differentiation [33], and the antimalarial drug dihydroartemisinin (DHA) at multiple time points over the course of 48-h post-treatment. While we found significant alterations in the immediate expression profile of *PfAP2-G* upon treatment with both effectors, they also significantly alter the *Pf* asexual developmental rate and stage composition (rings vs. trophozoite vs. schizont stages). This parameter can bias the mRNA expression pattern when measured by RT-qPCR. We next evaluated the potential involvement of lactate and kynurenic acid, two metabolites abundant in severe malaria patients [45, 46, 58, 59], in gametocyte gene regulation and found that neither of them significantly altered GRG expression under the assessed conditions. Our data emphasize a crucial parameter, parasite stage composition, which must be considered when interpreting the immediate response of GRG mRNA levels using RT-qPCR in malaria research, specifically when systematically screening for new regulators of this essential process for malaria transmission.

## Methods

### *Plasmodium falciparum* in vitro culture

*Pf* NF54 MR4-1000 *wt* cells were generously provided by the Malaria Research Reference Reagent Resource Center MR4. The *NF54-gexp02-Tom* line [19] was generously provided by the laboratory of Dr. Alfred Cortés, Barcelona Institute for Global Health (ISGlobal), Spain. *Pf* parasites were cultured in human RBCs using standard methods [60]. Briefly, parasites were grown in filtered flasks (LifeGene, #TCF012600S), at 4% hematocrit in pooled uninfected RBCs, provided by the Israel Blood Bank (Magen David Adom Blood Donations, Israel IRB #SMC-13-0954), and incubated at 37 °C in a gas mixture of 1% O_2_ and 5% CO_2_ in N_2_. Parasites were maintained in RPMI-1640 medium (Biological Industries, #11-100-1 M) supplemented with 25 mg/ml HEPES (Sigma-Aldrich, #H3375), 50 μg/ml hypoxanthine (Merck Millipore, #4010CBC), 2 mg/ml sodium bicarbonate (J.T. Baker, #144-55-8), 20 μg/ml gentamycin (Sigma- Aldrich, #G9654) and 0.5% Albumax II (Thermo Fisher Scientific, #11021-045). The *NF54-gexp02-Tom* line was grown in complete media supplemented with choline 2 mM (Sigma-Aldrich, #C7017) as reported [19] and removed only for gametocytogenesis induction. *Pf* growth was monitored using methanol-fixed, Giemsa-stained (Merck Millipore, #109203) blood smears [61]. *Pf* cultures were tested once a week for the presence of *Mycoplasma* using the MycoAlert™ PLUS kit (LONZA, #LT07-703).

### *Pf* culture synchronization

Ring-stage *P. falciparum* parasites were synchronized twice a week for regular maintenance and at least once prior to experiment plating using sorbitol lysis [62]. Briefly, *Pf* media were removed from *Pf* cultures and the blood pellet was incubated at 37 °C with D-sorbitol (Sigma-Aldrich, #S1876) 5% solution for 5 min. Immediately after, sorbitol was removed by centrifugation (1500 rpm, 5 min) and the blood pellet was washed twice with pre-warmed media. The blood pellet was resuspended in complete media and plated in new flasks.

### Method development in the robotic unit

Robot-automated RNA extraction, cDNA synthesis and RT-qPCR plating methods were developed for high-throughput RT-qPCR and programmed for a Biomek i5 Automated Workstation (Beckman Coulter) using its associated Biomek software version 5.0 (Beckman Coulter). To obtain dynamic setups for variable volumes and numbers of samples, input array worksheets were generated in Microsoft Excel 2016 using macros developed in Microsoft Visual Basic for Applications. These user-friendly input array worksheets enable the user to change the number, arrangement and organization of samples and volumes in the plates and automatically generate a.csv file compatible with and loadable to the Biomek i5 method.

### Robot-automated RNA extraction method

The robot-automated RNA extraction method was developed and calibrated for processing up to 96 samples simultaneously, based on the protocol of the RNAdvance Tissue® kit (Beckman Coulter, #A32646), which uses an extraction strategy based on magnetic beads adapted for automated workstations. The specific adaptations made to the protocol are the following:

40 µl of infected RBC with trophozoites and/or schizonts of parasitemia > 3.5% or 80 µl of infected RBC with rings of parasitemia higher than 4%, were defined as the input biological material per sample in each extraction well; 60 µl of lysis LBE^®^ Buffer + 3 µl of resuspended Proteinase K^®^ were used for lysis of 40 µl *Pf*-iRBC input. Alternatively, 120 µl of Lysis LBE^®^ Buffer + 6 µl of resuspended Proteinase K^®^ were used for lysis of 80 µl iRBC input; 175 µl of Binding BBC^®^ Buffer was used to bind the RNA. Three successive washes with 200 µl of washing WBD^®^ buffer for 40 µl *Pf*-iRBC input or 400 µl washing WBD^®^ buffer for 80 µl iRBC input were performed. Three successive washes with 200 µl 70% ethanol were carried out. DNase digestion was omitted in this step, and elution was carried out with 35 µl of ultra-pure water free of RNases and DNases per sample. After RNA elution, DNase digestion was performed according to the manufacturer’s manual, using the DNA-*free* Removal Kit (Invitrogen, #AM1906) for all experiments using RNA produced by the robot-automated method.

### Robot-automated cDNA synthesis method

The *Robot-automated cDNA Synthesis Method* was developed to dilute the extracted RNA with ultra-pure water into a final desired initial RNA amount, then mixing it with cDNA Master Mix solution in preparation for cDNA synthesis. This method was calibrated for processing up to 96 samples simultaneously and for final reaction volumes of 20, 40 or 60 µl. This method uses as input materials the purified RNA samples, ultra-pure water and a cDNA Master Mix prepared from the reagents of the High-Capacity cDNA Reverse Transcription Kit (Thermo Fisher Scientific, #4368814) according to the manufacturer’s instructions. The cDNA Master Mix is first aliquoted into the defined amount of reaction wells to achieve a 1:1 mixture with the diluted RNA. RNA is then aliquoted to the well assigned to each sample according to the desired input RNA quantity (varying between 100 to 1500 ng per reaction) and diluted to the desired reaction volume with ultra-pure water as calculated automatically in the input array worksheet.

### Robot-automated RT-qPCR plating method

The *Robot-automated RT-qPCR Plating Method* was developed and calibrated for plating 384-well plates for RT-qPCR, with a final reaction volume of 10 µl according to the defined locations in the input array worksheet. This method uses a diluted cDNA solution (1 µl of cDNA and 3.4 µl of ultra-pure water per reaction) and a SYBR Mix solution (5 µl of SYBR-fast Master Mix (Applied Biosystems) and 0.6 µl of forward + reverse primers, 10 µM mix per reaction) as input solutions for each sample and each gene. First, 5.6 µl of the SYBR Mix solution for each evaluated gene is aliquoted into each defined reaction well. Second, 4.4 µl of diluted cDNA solution is aliquoted into each one of the wells assigned to each sample.

A complementary method, robot-automated RT-qPCR aliquoting method, was also developed. This method prepares and aliquots the diluted cDNA solutions and the SYBR Mix solutions in the format needed by the robot-automated RT-qPCR plating method. Specifically, this method first aliquots the SYBR Mix solutions for each evaluated gene from an input 1.5-ml tube into an eight-microwell format. It then dilutes the cDNA samples with ultra-pure water (1 µl of cDNA and 3.4 µl of ultra-pure water per reaction) and aliquots each sample into a two-microwell format. Additionally, this method generates a standard curve after the last tube that contains sample cDNA under the parameters defined in its associated input array worksheet. The standard curve generated by this method is performed by mixing equal aliquots from the cDNA of each sample of the experiment (Standard 1) and then serially diluting this standard 1:5 five times consecutively to generate Standards 2 to 5. This method adds one extra sample with no cDNA template to run as a no-template control (NTC).

### RNA extraction, reverse transcription and RT-qPCR quality control culture treatment

For the experiments used as technical validation of the robot-automated methods, NF54 *Pf* parasites were cultured regularly, and RNA was extracted from high parasitemia cultures (> 3.5%) at the respective time points and stages indicated for each assay. Parasites were cultured in three technical culture replicates per biological repeat.

### RNA extraction

For the RNA extraction quality control compared with other established methods, three different RNA extraction methodologies were used: robot-automated magnetic binding (rMB), solid phase extraction (SPE) or liquid-liquid extraction (LLE). The protocol for the rMB method was carried out as described in the previous section. Extraction using SPE was carried out with the miRNeasy Mini Kit (Qiagen, #217004). LLE RNA extraction was performed using phenol:chloroform extraction with the BioTri RNA reagent (Bio Labs, #009010233100). The SPE and LLE methods were carried out according to the manufacturer’s protocol; 40 µl of *Pf*-iRBCs with ~ 3.5% trophozoite-stage parasites or 80 µl of *Pf*-iRBCs with ~ 5% ring-stage parasites was used as input sample for the three methods in three technical culture replicates of three independent biological repeats. After RNA extraction, DNase digestion was performed according to the manufacturer’s manual using the DNA-*free* removal kit (Invitrogen, #AM1906) or the RNase-Free DNase set (Qiagen, #79,254) only for the experiments in which the miRNeasy Mini Kit was employed.

Quality control assessment of the robot-automated RT-qPCR system and the screenings for evaluation of gene expression in response to DHA, choline, lactic acid and kynurenic acid were performed with RNA obtained using the robot-automated RNA extraction method, using a standardized input of 80 µl of *Pf*-iRBC regardless the parasitic stage to ensure enough RNA was extracted for afterwards cDNA synthesis and RT-qPCR. DNase digestion was performed according to the manufacturer’s manual, using the DNA-*free* Removal Kit (Invitrogen, #AM1906).

### RNA quantification and purity assessment

Purified and DNase-treated RNA was quantified spectrophotometrically using a NanoDrop 8000 UV spectrophotometer (Thermo Fisher Scientific). The A_260_/A_280_ and A_260_/A_230_ purity ratios were also obtained with this instrument.

### RNA integrity electrophoresis

RNA integrity was estimated by electrophoresis in 1% agarose gel prepared in TAE 1 × buffer with ethidium bromide. RNA sample was loaded with 6 × DNA loading dye (Thermo Fisher Scientific, # R0611), adjusted to 1 × concentration with ultra-pure water, and the GeneRuler 1 Kb DNA Ladder molecular weight marker (Thermo Fisher Scientific, # SM0311) was used as a reference. Electrophoresis was run for 45 min at 200 V to allow proper separation of the RNA.

### RNA integrity tapestation analysis

RNA integrity was assessed using a TapeStation 2200 RNA ScreenTape (Aligent Technologies) capillary electrophoresis system. The RNA Integrity Number (RIN) was calculated using the TapeStation Analysis Software A.02.02 and analyzed for eukaryotic RNA samples.

### cDNA synthesis

cDNA was synthetized by reverse transcription (RT) using the robot-automated cDNA synthesis method, developed in-house as previously described, and an equalized initial amount of 450–500 ng RNA per sample. The reverse transcription reaction was carried out in a SimpliAmp thermocycler (Thermo Fisher Scientific) according to the High-Capacity cDNA Reverse Transcription Kit (Applied Biosystems) conditions as specified in the manual.

### RT-qPCR quality control and gene expression analysis

Gene expression of *Pf* early gametocytogenesis markers was evaluated by quantitative reverse-transcription PCR (RT-qPCR). After treatment induction, RNA extraction and cDNA synthesis, 384-well microplates for RT-qPCR (Axigen, #PCR384M2-C) were plated using the robot-automated RT-qPCR plating and RT-qPCR Aliquoting methods, as described in the previous sections. The RT-qPCR reactions were carried out in a QuantStudio 6 Flex Real-Time System (Applied Biosystems) using the default program for SYBR-fast reagents.

The sequences of all the gametocytogenesis genetic markers and reference genes used for normalization and RT-qPCR gene expression analysis are summarized in Additional file 1: Table S1. For RT-qPCR quality control validation, standard curves from all the genes were generated by the automated robotic unit in three biological replicates of RNA extracted from synchronized ring- or trophozoite-stage parasites. The statistical parameters [slope, correlation coefficient (r^2^) and efficiency] of the generated standard curves were calculated as reported [63] and verified to comply with the expected values in published guidelines [63, 64] within each biological replicate (Additional file 2: Table S2) and for the average of the three biological replicates (Additional file 3: Table S3).

For gene expression analysis, expression levels of gametocyte markers under all conditions were normalized to the expression of the ubiquitin-conjugating enzyme (*uce*, PF3D7_0812600) [21], given its increased stability compared to the other reference genes [63]. For all gene expression analyses, transcript level quantification was carried out using the standard curve method in the QuantStudio Real-Time Software (Applied Biosystems). Relative quantification using the standard curve method was chosen in this study as it is recommended for *Pf* because of its demonstrated higher robustness as compared to the ΔΔCt method [63], especially since it does not require the primer efficiencies to be strictly similar. Data in gene expression analyses are represented as the transcript levels of the analyzed gene/transcript levels of *uce*, both quantified with their respective standard curves on the same sample. The data represent the mean of a total of nine technical repeats (3 technical replicates of the RT-qPCR reaction within 3 technical replicates of the biological treatment) within a minimum of three independent biological replicates.

### SPUD assay

The SPUD assay was performed as reported [65]. Briefly, 100 µM stock SPUD template (Integrated DNA Technologies) was serially diluted 1:10 and an initial RT-qPCR was carried out to choose the optimal dilution for amplification comparison. A working concentration of 10^− 5^ nM (10^th^ dilution in the series) with a Ct of approximately 25 was chosen. Each RT-qPCR reaction well was prepared by the robotic unit by mixing the following: 1 µl of 10^− 5^ nM SPUD template, 0.6 µl of 10 µM SPUD forward and reverse primers mix (Integrated DNA Technologies) [65], 1 μl of cDNA previously prepared from iRBC (rings of trophozoites) using the robot-automated methods, 5 µl of SYBR-fast Master Mix (Applied Biosystems) and completing to a final reaction volume of 10 µl with ultra-pure water. The *Pf*-derived cDNA used in this assay as a spectator template does not contain sequences that can amplify with the SPUD primers. Negative inhibition control was prepared by replacing the spectator cDNA with ultra-pure water, and positive inhibition control was prepared by replacing the spectator cDNA with phenol. After amplification, the Ct values of all samples were compared in order to identify potential reaction inhibition.

### No reverse-transcriptase assay

RNA extracted from *Pf*-iRBCs using the robotic method was treated with DNase, as described in the relevant section above. Synthesis of cDNA was performed using the DNase-treated RNA samples as described, with or without reverse transcriptase. The output was used in RT-qPCR amplification and Ct values for each sample were compared to validate the absence of DNA after DNase treatment.

### Dihydroartemisinin (DHA) treatment

Treatment with dihydroartemisinin (DHA) was carried out as reported previously [35]. Synchronized NF54 *Pf* trophozoite-stage parasites were plated at an initial parasitemia of 2% and cultured for 48 h under the following conditions: complete media (non-treated control), DHA 5 nM or 10 nM, or 0.1% (v/v) DMSO (solvent control). Three hours post-initiation of the experiment, media from all samples were removed and the cells resuspended in fresh RPMI complete media. Then, *Pf*-iRBCs (at least 2% parasitemia) were harvested directly from the culture at defined time points (4, 8, 12, 25, 30 and 48 h) for robot-automated RNA extraction.

The same experimental setup was repeated with choline treatment. Synchronized NF54 *Pf* trophozoite-stage parasites were cultured for regular maintenance in complete media supplemented with 2 mM choline and plated at 2% initial parasitemia with or without 10 nM DHA. Three hours post-induction, media from all samples were removed, and the cells resuspended in fresh RPMI complete media. Twenty-four hours post-induction, *Pf*-iRBCs were harvested directly from the culture at the defined time points for robot-automated RNA extraction.

### Gametocytogenesis transcriptional profile under choline treatment

To generate early temporal gametocytogenesis gene expression profiles, *Pf* parasites were cultured in the presence or absence of 2 mM choline. Parasites from the *NF54-gexp02-Tom* line were used, given their proven sensitivity to choline treatment [19] and capacity to evaluate %GCR by flow cytometry. Briefly, *NF54-gexp02-Tom* parasites cultured constitutively in complete RPMI media supplemented with 2 mM choline were sorbitol-synchronized in the ring stage and plated 24 h later as 2% early trophozoites in RPMI media (- choline) or media supplemented with 2 mM choline (+ choline) for a total of 48 h (one complete intraerythrocytic cycle). Cells were harvested and RNA extracted at 0, 6, 12, 18, 24, 30, 36 and 48 h post-initial plating for cDNA synthesis and RT-qPCR gene expression analysis.

### Parasite growth assays by flow cytometry

Synchronized trophozoite stage parasites at 1.0% parasitemia were cultured for 3 h under the following conditions: DHA in concentrations of 5 nM or 10 nM, or DMSO in concentrations of 0.05% or 0.1% (solvent controls). Parasites treated with complete media were used as non-treated control. DHA was washed and media were replaced 3 h post-induction. Parasitemia was monitored every 24 h using flow cytometry analysis as reported [66]. Cells were stained for 20 min at 37 °C with staining solution containing 2 μM Hoechst-33342 (Invitrogen, #H1399) and 10 ng/ml thiazole orange (Sigma-Aldrich, #390062) in PBS (Biological Industries, #02-0230-1A). Analysis was performed using a ZE5™ flow cytometer (Bio-Rad) equipped with 355 and 488 nm lasers. Gating for infected cells was based on uninfected cells (uiRBCs) stained under the same conditions.

The impacts of choline on parasitic growth and on the percentage of gametocyte conversion rate in the *NF54-gexp02-Tom* line were also determined by a flow cytometry growth assay in a fashion similar to that used for DHA. In this case, cells were stained using 2 μM Hoechst-33342 in PBS, and analysis was performed by the 488 and 581 nm laser channels for assessing Hoechst-33342 and the fluorescent protein tdTomato, respectively. The percentage of *gexp02*^+^ iRBC (a proxy for the percentage of sexually committed cells) was calculated automatically by the ZE5™ flow cytometer software as the percentage of the double-positive population (Hoechst-33342^+^ and *gexp02*^+^) as compared to the single positive, for the Hoechst-33342 DNA stain only.

### Parasite growth assay and stage composition analysis in Giemsa-stained smears

The flow cytometry growth assay performed for DHA was confirmed using manual parasitemia quantification in Giemsa-stained smears. Blood smears were taken every 24 h for 72 h and stained with Giemsa solution. Parasitemia was quantified in 10 microscopic fields per treatment per time point (3 biological replicates in total) and the percentage of iRBC calculated over the total amount of RBC.

A stage composition analysis was performed using Giemsa-stained smears. Smears were taken at each indicated time point and the parasites classified as ring, trophozoite or schizont stage according to microscopic morphology, excluding unviable, pyknotic and dead parasites. The abundance of each parasitic developmental stage (ring trophozoite or schizont stage) was calculated as the percentage of the parasites in each stage relative to the total amount of parasites, counting at least 50 *Pf*-iRBCs in at least 10 different microscopic fields for each technical repeat of each biological replicate.

Merozoite productivity during schizogony was estimated by quantifying the average number of daughter cells (merozoites) in 10 representative late schizonts in each of the three biological replicates at the 12 h time point for all treatments.

### Chloroquine treatment

NF54 *Pf* ring-stage parasites were plated at an initial parasitemia of 5% and cultured for 24 h with complete RPMI media (non-treated control) or chloroquine 40 nM (Sigma Aldrich, C6628). Trophozoite-stage parasites were plated at an initial parasitemia of 5% and cultured for 4 h with complete RPMI media (non-treated control), or 40 nM or 80 nM chloroquine. After induction, *Pf*-iRBCs were harvested directly from the culture at the defined time points for robot-automated RNA extraction.

### Lactic acid and kynurenic acid treatment

Synchronized NF54 *Pf* trophozoite-stage parasites were cultured for 16 h in complete media with or without 2 mM choline supplementation and the following treatments: non-treated control, sodium DL-lactate 5 mM [43] (Sigma Aldrich, #71720) or kynurenic acid 250 nM (Sigma Aldrich, #K3375). *Pf*-iRBCs were then harvested directly from the culture for robot-automated RNA extraction.

### Sexual conversion assay by gametocyte culture

The effects of DHA in sexual conversion were analyzed by monitoring gametocyte cultures as previously reported [35]. Briefly, sorbitol-synchronized NF54 *Pf* ring-stage parasites were diluted to an initial parasitemia of 1.5% and 22 h after synchronization (day 0) were treated with dihydroartemisinin 10 nM or DMSO 0.1% (solvent control). Media was replaced 3 h later and 24 h later. Media were replaced daily with RPMI without Albumax-II and supplemented with 10% human serum (Sigma Aldrich, #H4522) and N-acetylglucosamine (NAG, Sigma Aldrich, #PHR1432) 50 mM to prevent asexual stage proliferation.

Giemsa-stained smears were taken daily for manual microscopic quantification. Asexual stage parasitemia (percentage of iRBC/total RBCs) on day 1 and gametocytemia (percentage of gametocytes/total RBCs) on day 7 were manually quantified over 10 microscopic fields per treatment in three independent biological replicates. The percentage of sexual conversion was calculated as reported [35], dividing the total gametocytemia on day 7/parasitemia on day 1 and multiplying by 100%.

### Statistical analyses

All statistical analyses performed in the present study were carried out on the mean of at least three independent biological replicates using the GraphPad Prism® version 9 software or R version > 4, as indicated in each figure legend. In all cases, the mean of each set of biological repeats is represented; individual values of each biological repeat are represented as scatter plots and standard deviation as error bars. Multiple comparisons were performed where needed as indicated in the respective figure legends, and *p*-values were calculated to show statistical significance.

## Results

### A high-throughput automated RNA extraction method yields high-quality RNA

We developed a high-throughput platform to robustly screen mRNA levels and monitor gene expression profiles of *Pf*-infected RBCs (*Pf*-iRBCs), particularly to determine the immediate transcriptional responses of *Pf* sexual markers. A robot-automated technology, such as the Biomek i5 Automated Workstation used here, enables the use of very low volumes of *Pf*-iRBCs to process numerous samples simultaneously. Importantly, we established a platform with modular programmed methods for this robotic workstation, which consists of three sequential, ‘hands-free’ steps: (i) RNA extraction, (ii) cDNA synthesis and (iii) RT-qPCR plating. To ensure the suitability of the extracted RNA for the RT-qPCR assays, the yield and quality of the extracted RNA obtained using our robot-automated magnetic binding (rMB) method were determined. We compared our rMB method to two widely used RNA methods: the liquid–liquid solvent extraction method (LLE) [67] and the solid-phase extraction method (SPE), which employs silica columns [21].

**Fig. 1:**
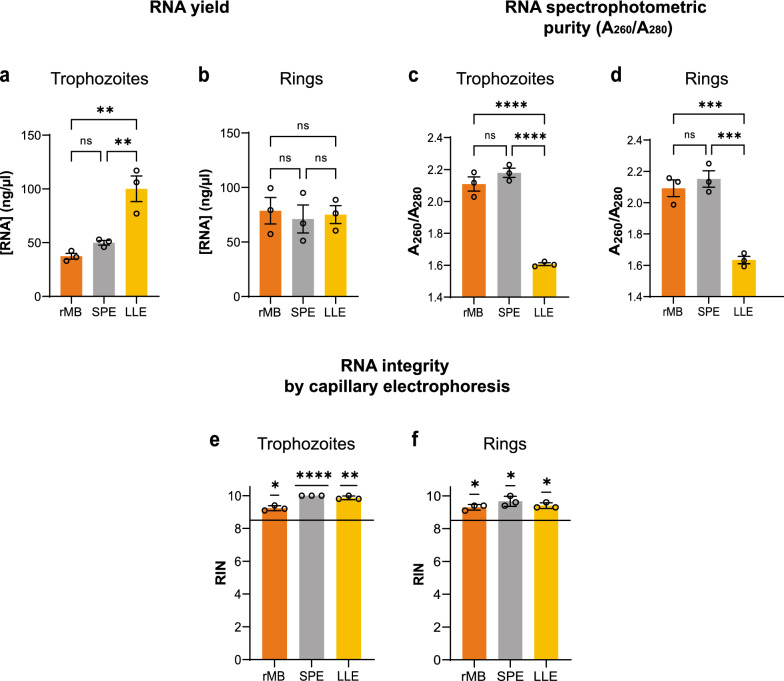
The robot-automated method yields high purity *Pf* RNA. **a**, **b** Spectrophotometric quantification of the RNA yield extracted from *Pf*-iRBC ring or trophozoite stage cultures using (i) robot-automated magnetic binding (rMB), (ii) solid phase extraction (SPE) or (iii) liquid–liquid extraction (LLE) methods. **c**, **d** Mean A_260_/A_280_ purity ratio determined spectrophotometrically for samples extracted with the same methods. **e**, **f** Mean RNA integrity number (RIN) calculated by capillary electrophoresis (TapeStation^®^ analysis). Panels *a*–*d* present the mean of three biological replicates (*n* = 3) with three technical replicates each. Panels **e**, **f** present the mean of three biological replicates (*n* = 3) with one technical replicate each. Error bars represent SD. A one-way ANOVA with Tukey’s multiple comparisons test was performed for panels *a*–*d*, and a single-value t-test of the mean RIN against the RIN value of 8.5 (considered minimum for high-quality RNA [71], represented as a horizontal line in the plot) was performed for panels **e**, **f**. ns = *p* ≥ 0.05 (non-significant), *p < 0.05, **p ≤ 0.01, ***p ≤ 0.001 and ****p ≤ 0.0001

RNA was extracted using each method (rMB, LLE and SPE), and UV spectrophotometry was used to assess the quantity and quality of the extracted RNA (Fig. 1a–d). We show that when using a minimal *Pf*-iRBC input (40 µl for trophozoite-stage parasites and 80 µl for ring-stage parasites), the yield of total RNA extracted from *Pf*-iRBCs using our rMB method is similar to the yield obtained using the SPE method (Fig. 1a, b). The LLE method seemed to yield a significantly higher RNA concentration than the two other methods. However, measurements of the A_260_/A_280_ purity ratio correlated to protein impurities in the extracted RNA [68], demonstrating that the rMB and SPE methods exhibit the highest RNA purity within the ideal range of 2.0–2.2 for pure RNA samples accepted in the field [68] (Fig. 1c, d). Conversely, the LLE method yielded significantly lower purity, indicative of high levels of protein contamination [68].

The A_260_/A_230_ ratio was found to be below the ideal value of 1.8 [68] when extracting RNA from ring or trophozoite *Pf*-iRBCs with all three methods (Additional file 4: Fig. S1). It is known that the A_260_/A_230_ ratio can be affected by numerous contaminants, such as guanidinium salts, ethanol, isopropanol and detergents, which are common reagents used in RNA extraction methods [68]. Furthermore, these spectrophotometric purity parameters have been demonstrated to be highly variable, and thus unreliable for samples with low RNA concentrations [68]. Of note, according to the current MIQE guidelines for RT-qPCR assays [64], reporting the A_260_/A_230_ ratio of RNA is not considered to be essential information. Nevertheless, we performed two additional assays (capillary electrophoresis and agarose gel electrophoresis) to assess the quality of the extracted RNA.

First, we assessed RNA integrity via agarose gel electrophoresis, and the results indicated non-degraded RNA samples [69, 70] (Additional file 5: Fig. S2). We further assessed RNA integrity by capillary electrophoresis. The RNA integrity number (RIN) was determined for a selected number of samples from each biological replicate (Fig. 1e, f). A RIN value of 8.5 is considered as the lower limit for high-quality RNA integrity [71, 72]. Here, we show that high RIN values were obtained for each of the three RNA extraction methods (Fig. 1e, f), indicating a high-quality integral RNA suitable for downstream RT-qPCR applications [71, 72]. Representative capillary electropherograms of samples extracted from each method are presented in Additional file 6: Fig. S3.

Collectively, these four different analyses demonstrate that the RNA extracted using our rMB method is comparable in yield, quality and integrity to traditionally accepted RNA extraction methods frequently used in gene expression studies in malaria [12, 21, 35].

### The automated RT and RT-qPCR methods meet optimal requirements for gene expression analysis

**Fig. 2:**
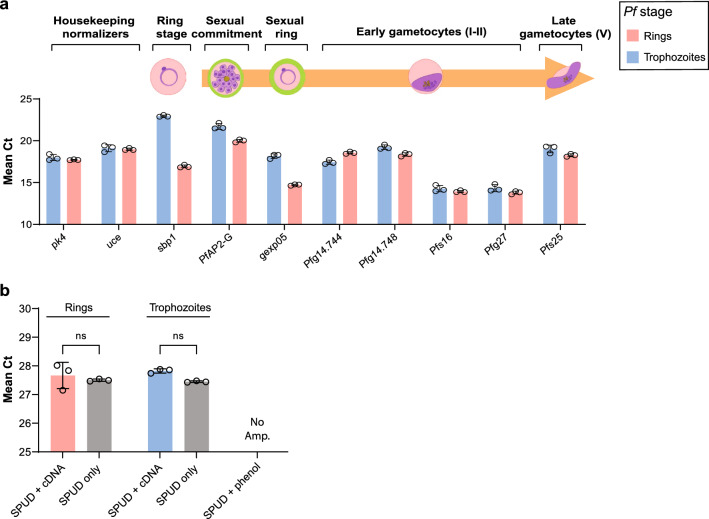
The robot-automated method enables precise gene expression analysis by RT-qPCR. **a** Gene panel selected to evaluate early gametocytogenesis-related gene (GRG) expression dynamics. The basal expression of each gene in the panel was evaluated for ring- and trophozoite-stage cultures, which continuously commit and generate a basal level of gametocytes expressing these genes. Technical repeatability was evaluated by the standard deviation value (error bars) of the Ct in three independent biological repeats (*n* = 3). **b** SPUD polymerase inhibition assay for cDNA synthesized by robot-automated methods from ring- and trophozoite-stage parasites. Artificial SPUD template at a 10^− 5^ nM concentration was used as a non-inhibition control, and phenol was used as a positive inhibition control. No Amp. = no amplification (*Ct* ≥ 40). *a*–*b* Mean of three biological replicates (*n* = 3) with three technical replicates each. Error bars represent SD. A two-way ANOVA with Šidák’s multiple comparisons test was performed for Panel *b*. ns = *p* ≥ 0.05 (non-significant)

Having established the reliability of our RNA extraction method, we could direct our automated platform towards the downstream profiling of gene expression in *Pf*-iRBCs. Importantly, this platform enables us to systematically monitor a large-scale number of samples (up to 96 simultaneously), with minimum manual intervention, a major advantage when compared to the standard *Pf*-iRBC RNA extraction methods currently employed in the field. Thus, we proceeded to generate automated protocols for two additional sequential steps: cDNA synthesis (reverse transcription, RT) and RT-qPCR. Since sexual commitment is an essential process in malaria transmission, we decided to apply our system to evaluate the expression of a selected panel of early gametocytogenesis-related genes (GRGs) (Fig. 2a). The RT-qPCR panel used for the analysis included a total of 10 parasite genes: two *Pf* reference genes, the protein kinase 4 *pk4* and the ubiquitin-conjugating enzyme *uce* [21], the gametocytogenesis master regulator *PfAP2-G* [12], the sexual ring marker *gexp05* [18], four early gametocyte markers, *Pfg14.744, Pfg14.748, Pfs16* and *Pfg27* [17, 73], the mature gametocyte marker *Pfs25* [74] and the ring-stage marker *sbp*1 [75]—to serve as a non-gametocyte control.

To evaluate the technical repeatability of the RT-qPCR assay [64], we extracted RNA from either synchronized trophozoite-stage or ring-stage *Pf*-iRBCs. We then synthesized cDNA and performed RT-qPCR to estimate the basal expression levels of each of these genes on three independent biological replicates and, in each, three technical replicates. It is important to note that, although the predominant parasite forms in the cultures (rings or trophozoites) are asexual stages, a basal number of these asexual parasites continuously commit to sexual development and produce gametocytes. Thus, this existing low level of committed asexual and developing sexual parasites is responsible for the observed expression of gametocyte-specific transcripts [20, 76]. The Ct values of the amplification plots of the gene panel showed clear repeatability and reproducibility [64], as demonstrated by the standard deviation values of the replicates. Notably, these values were mostly smaller than one amplification cycle (Ct) between the three independent biological replicates for each *Pf* blood stage (Fig. 2a). Correspondingly, the standard curves applied for quantification of this gene panel showed optimal efficiencies (between 90–100%) and slopes for accurate quantification, as well as correlation coefficients (r^2^) of at least 0.99 (a representative example from one biological replicate is presented in Additional file 2: Table S2).

As a measure of reproducibility, the standard curves for each gene were plotted for three independent biological replicates of ring- and trophozoite-stage parasites (Additional file 7: Fig. S4) and the statistics applied to their means (mixed model ANOVA for batch effect). Both the efficiency and r^2^ of the average of the standard curves were also close to optimal values, and data dispersion was minimal (statistical parameters of the mean of the standard curves are presented in Additional file 3: Table S3), confirming the high level of reproducibility of the RT-qPCR standard curves. Analysis of the melting curve of the amplification product of each pair of primers in the gene panel showed one single discrete peak in the derivative of the fluorescence (Additional file 8: Fig. S5), demonstrating the absence of unspecific amplification products and primer specificity as per the accepted guidelines [64].

We further performed a No Reverse-Transcriptase (NRT) control assay , which enabled us to validate the absence of residual gDNA, post-RNA extraction (Additional file 9: Fig. S6). We extracted RNA using our rMB method from trophozoite-stage samples. During cDNA synthesis, reverse transcriptase was either added as per the protocol (RT-treated, yellow bars) or omitted (Non RT-treated, green bars). The cDNA synthetized from each sample was then used for RT-qPCR amplification as well as a no-template control (NTC, gray bars). As observed, the RT-treated samples presented Ct values well below the Ct threshold of 35, indicating positive amplification. However, the Non RT-treated samples and the no-template control presented no significant levels of amplification (Ct > 35 considered negligible [63]). These data indicate that there was no significant gDNA contamination in the DNAse-treated RNA samples, which could interfere with the RT-qPCR reaction cycles.

It is important to clarify that for several genes from our selected gene panel, the no-template control showed a weak amplification curve in some of the technical repeats with Ct values around cycle 35. This was likely due to the presence of primer dimers caused by multiple AT pairs in their sequence (presented in Additional file 1: Table S1), a common feature of malaria gene primers, as expected in cases of extremely high genomic AT content (more than 80% in coding sequences) [77]. This structural bias makes it technically challenging to generate highly sequence-specific primers that do not generate background fluorescence when using SYBR-green amplification. However, these low amplification curves were not observed consistently, even within the technical replicates of the no-template controls. Additionally, none of them appeared as considerable unspecific products in the melting curve analysis of the primers (Additional file 8: Fig. S5).

To determine potential contaminations originating from previous steps (i.e. RNA extraction, cDNA synthesis) that could inhibit the catalytic activity of the DNA polymerase during the RT-qPCR reaction, the SPUD assay is a reliable and quantitative quality control system [64]. Thus, a SPUD assay was carried out using cDNA prepared with our methods following RNA extraction from rings or trophozoite *Pf*-iRBCs. No statistically significant difference was observed between the mean Ct value of the SPUD amplicon alone or when the reaction is carried out in presence of spectator cDNA prepared from *Pf*-iRBCs (Fig. 2b), indicating absence of DNA polymerase inhibitors in the samples that could interfere with the RT-qPCR amplification. Phenol was used as a positive control for DNA polymerase inhibition [65], and no amplification of the SPUD template was observed in the presence of phenol (Fig. 2b).

Together, these data demonstrate the high robustness and reproducibility of the developed robotic methods and validate their effectiveness in RT-qPCR gene expression monitoring.

### Treatment with DHA alters the *PfAP2-G* expression profile and affects *Pf* asexual stage composition

Once we ensured that our RT-qPCR platform met the accepted quality standards, we aimed to exploit this high-throughput system to monitor how GRGs modify their temporal expression pattern under a condition previously suggested to induce sexual conversion. The antimalarial drug dihydroartemisinin (DHA) has been previously reported to induce sexual conversion when applied at sublethal concentrations [35]; hence, DHA was selected as a reference treatment. Since trophozoites have been shown to be more responsive to gametocytogenesis induction [20, 35], we examined the effect of DHA on the expression profile of selected genes of our panel when applied for a short pulse (3 h) during the trophozoite stage at sublethal concentrations.

**Fig. 3:**
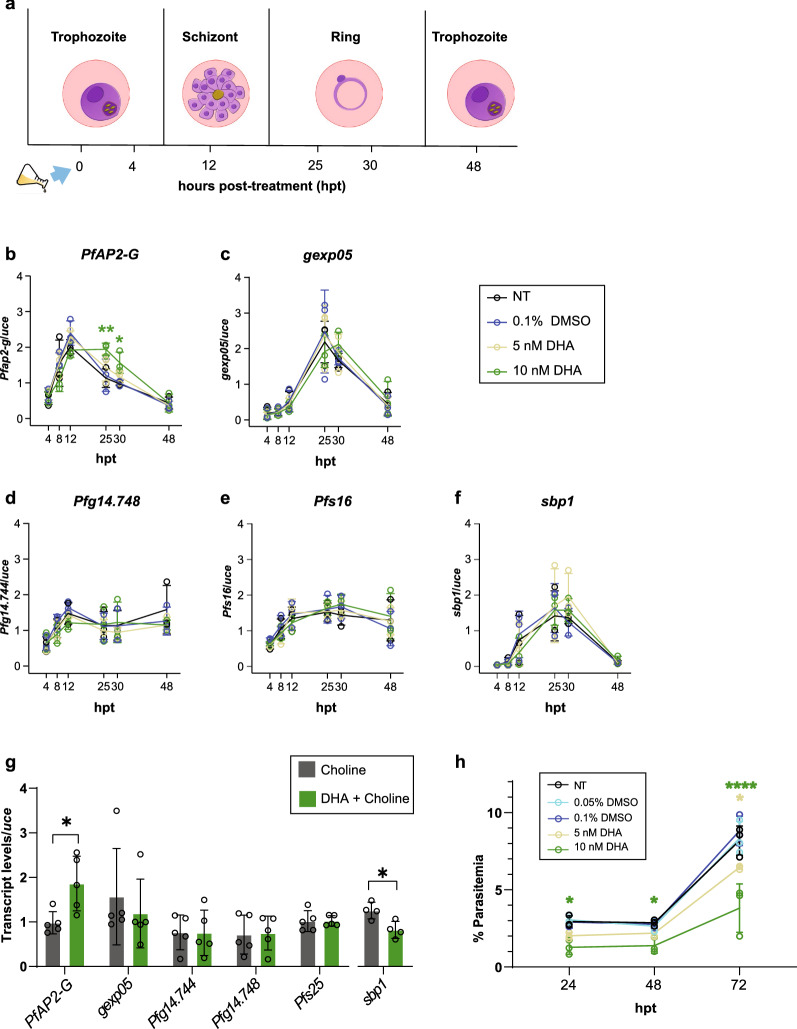
Dihydroartemisinin (DHA) alters the *PfAP2-G* expression profile and inhibits parasitic development. **a** Schematic illustration of the experimental setup: the transcriptional profiles of five GRG markers were built for synchronized *NF54* trophozoite-stage parasites treated over the course of one 48-h cycle after a short (3-h) pulse with DHA (5 or 10 nM) or 0.1% DMSO (solvent control) or non-treated (NT) control. The flask and arrow represent treatment induction starting at the first time point (0 hpt). RNA was extracted by the robotic unit at 4, 12, 25, 30 and 48 hpt for RT-qPCR expression analysis. The predominant stage in each time span is also presented in the illustration for comparison with the transcriptional profiles. **b–f** Temporal transcriptional profiles of the early gametocytogenesis markers *PfAP2-G* (**b**), gexp05 (**c**), *Pfg14.748* (**d**), *Pfs16* (**e**) and *sbp1* (**f**, a ring-stage marker). **g** Transcript levels of *Pf* GRGs: *PfAP2-G, gexp05, Pfg14.744, Pfg14.748, Pfs25* and *sbp1*, 24 h post-DHA induction under 2 mM choline treatment. **h** Growth assay for NF54 *Pf*-iRBCs post-treatment with DHA (5 nM or 10 nM) for 3 h (trophozoite stage), monitored by flow cytometry. The parasitemia was quantified every 24 h over a 72-h period. The respective volumes of DMSO (0.05% and 0.1%) were used as solvent controls, and non-treated (NT) parasites were used as negative control. The flow cytometry gating strategy for a representative example can be observed in Additional file 14: Fig. S11. In Panels **b**–**f** and **h**, data represent the mean of three independent biological repeats (*n* = 3) with three technical repeats each, while in Panel **g**, data represent the mean of five independent biological repeats (*n* = 5) for all the genes except for *sbp1* (4 biological repeats, *n* = 4). Mean transcript levels were calculated using the relative standard curve method and normalized to the transcript levels of *uce* in Panels b–f. Error bars represent SD. Two-way ANOVA with post-hoc tests were run using estimated marginal means with the R package 'emmeans' for Panels **b**–**f** and **h**. An unpaired *t*-test was performed between the DHA-treated and non-treated parasites for each gene individually in Panel **g**. *p < 0.05, **p ≤ 0.01 and ****p ≤ 0.0001

We thoroughly evaluated the temporal expression profile of *Pf* early GRG markers (*PfAP2-G, gexp05, Pfg14.748, Pfs16*, with *sbp1* used as an internal control) over the course of 48-h post-treatment (hpt) at six different time points: 4, 8, 12, 25, 30 and 48 h (Fig. 3a). We observed a significant change in the expression pattern of *PfAP2-G*, the key gametocytogenesis regulator gene, at 25 hpt and 30 hpt when treated with the highest concentration of 10 nM DHA (Fig. 3b). As observed, *PfAP2-G* expression pattern peaked at 12 hpt for all samples (including the solvent control and markers treated with the low concentration of DHA) but rapidly decreased in the time points that followed, except for the samples treated with 10 nM DHA. Under this treatment, the parasites continued to express high levels of *PfAP2-G* even at 25 hpt, and the mRNA levels only started to decrease at 30 hpt and beyond (Fig. 3b). These results support a previous report demonstrating the induction of sexual commitment gene expression by DHA at sublethal concentrations [35].

Pretreatment of parasite cultures with choline, a known gametocytogenesis repressor [9, 33], has been used in previous studies to maintain parasites in a non-induced state and evaluate the effect of treatments instead of constant gametocytogenesis induction in the absence of choline [35]. In our study, treatment with choline to maintain parasites in non-inducing conditions was not required to observe changes in the pattern of *PfAP2-G* expression (Fig. 3b). These results suggest that our platform could provide an improved resolution, enabling detection of small changes in gene expression even in inducing conditions in absence of choline as well as a *PfAP2-G* induction in a different experimental setup than previous reports [35].

While an alteration in *PfAP2-G* expression was observed under the DHA treatment, we could not detect alterations in its target genes, *Pfg14.744* and *Pfs16,* or in the sexual ring marker *gexp05,* over the course of 48 h (Fig. 3c–e). Thus, to determine whether pretreatment of the parasites with choline would increase our method’s ability to detect alterations in GRGs other than *PfAP2-G* by repressing the basal sexual commitment [19, 35], we cultured parasites in the presence of choline. *Pf*-iRBCs were pretreated with 2 mM choline for a minimum of two intraerythrocytic cycles; then, trophozoite-stage parasites were treated with a short (3 h) pulse of 10 nM DHA, and GRG expression was measured 24 hpt (Fig. 3g). Notably, we could detect a significant elevation in the *PfAP2-G* transcription level under DHA treatment. However, no significant changes were detected in the expression of *PfAP2-G* target genes, *gexp05*, *Pfg14.744* and *Pfg14.748* (Fig. 3g).

Importantly, DHA treatment resulted in a significant decrease in the expression level of *sbp1*, a highly specific ring-stage marker [6], when compared to the no drug control. This suggests either a delayed intraerythrocytic growth of the asexual forms in the DHA-treated samples or an increased number of parasites that converted into gametocytes (and consequently a reduced number of asexual parasites detectable by *sbp1*). This encouraged us to conduct a *Pf* growth assay combined with a blood stage composition analysis (rings vs. trophozoites vs. schizonts) under DHA treatment as well as a gametocyte culture assay to directly quantify gametocyte abundance by Giemsa smears after DHA treatment (Fig. 3h, Additional file 10: Fig. S7 and Additional file 11: Fig. S8). NF54 trophozoite-stage *Pf*-iRBCs were treated with DHA for 3 h, and FACS analyses combined with microscopy counting of Giemsa-stained smears were used to evaluate the effect of DHA on parasitic growth dynamics. As expected, exposure to sublethal concentrations of DHA (for 3 h) decreased parasite growth as early as 24 h post-treatment (upon the first RBC invasion). These results are in alignment with previously reported studies [35]. In our study, the detrimental effect of DHA was further observed during the second RBC invasion cycle (72 hpt). Moreover, stage composition analysis revealed delayed intraerythrocytic parasite development (Additional file 10: Fig. S7b), with a significantly smaller proportion of ring-stage parasites observed in the DHA-treated samples when compared to the non-treated control. These findings are also in accordance with previous published reports [78].

Furthermore, microscopic evaluation of gametocyte abundance after DHA treatment did not reveal a significantly increased proportion of sexual parasites (Additional file 11: Fig. S8), which supports the notion of the observed decrease in *sbp1* expression being due to asexual growth delay and not to a significant contribution of increased commitment into the sexual stage. This result is in line with a previous report where DHA treatment did not significantly increase sexual commitment in inducing conditions (choline depletion) [35], suggesting that DHA treatment alone in inducing conditions (choline-depleted cultures) may initially alter the expression of *PfAP2-G*, but not necessarily produce an increased gametocyte abundance as opposed to choline supplementation, where an increase in sexual commitment after DHA treatment was previously reported [35].

Thus, the observed significant differences in asexual parasite stage composition may contribute, at least partially, to the detected differences in the expression dynamics of *PfAP2-G* (Fig. 3b). Indeed, under DHA treatment, *PfAP2-G* expression remained elevated for a longer period and peaked between 12 and 30 hpt (Fig. 3b), coinciding with a significantly lower percentage of ring-stage parasites (Additional file 10: Fig. S7b) but no net increase in gametocyte abundance (Additional file 11: Fig. S8). Since mature schizonts express *PfAP2-G* at high levels [21], our data suggest that changes in asexual stage composition caused by DHA can be responsible for the observed increase in *PfAP2-G* mRNA levels as detected by RT-qPCR; thus, an isolated change in *PfAP2-G* levels needs to be cautiously interpreted, in light of analysis of both asexual and sexual populations.

We next monitored the expression profiles of GRG under treatment with sub-lethal doses of chloroquine (CQ), another pertinent antimalarial drug. In this case, no significant alterations in sexual gene expression were observed (Additional file 12: Fig. S9), in line with previous findings [35, 40].

Overall, our findings emphasize the complexity in monitoring immediate responses in gametocyte markers (within a period of 48 h) and the importance of different experimental settings to evaluate gametocyte commitment (inducing or non-inducing conditions by choline supplementation/Kennedy pathway activation) and highlight the need to combine complementary approaches to interpret GRG expression changes.

### Choline treatment represses *PfAP2-G* expression and affects *Pf *stage composition by increased growth

We showed that DHA treatment induced *PfAP2-G* expression as well as modified parasite growth and stage transition. Therefore, we decided to examine the expression profiles of *Pf* GRG using a gametocyte induction method that avoids stress on parasitic development. Choline is a key metabolite in the Kennedy pathway, a metabolic pathway active in malaria parasites and central to the formation of cellular membrane phospholipids during schizogony [34]. It has been previously reported that the presence of choline inhibits gametocyte formation [33] but does not inhibit parasitic growth [34]. Accordingly, we used our system to monitor the expression pattern of a selected number of GRG in choline-treated *Pf* cultures, following its depletion in the trophozoite stage. The *NF54-gexp02-Tom* fluorescent *Pf* line [19], previously used to evaluate both GRG expression by RT-qPCR and the percentage of gametocyte commitment rate (%GCR) [35] by flow cytometry, was used. This line expresses a fluorescent reporter under the control of the *gexp02* promoter, a highly specific early gametocyte marker and a target of *PfAP2-G* [13, 20]*.* The expression of the fluorescent marker begins early on at the sexual ring stage [19].

**Fig. 4:**
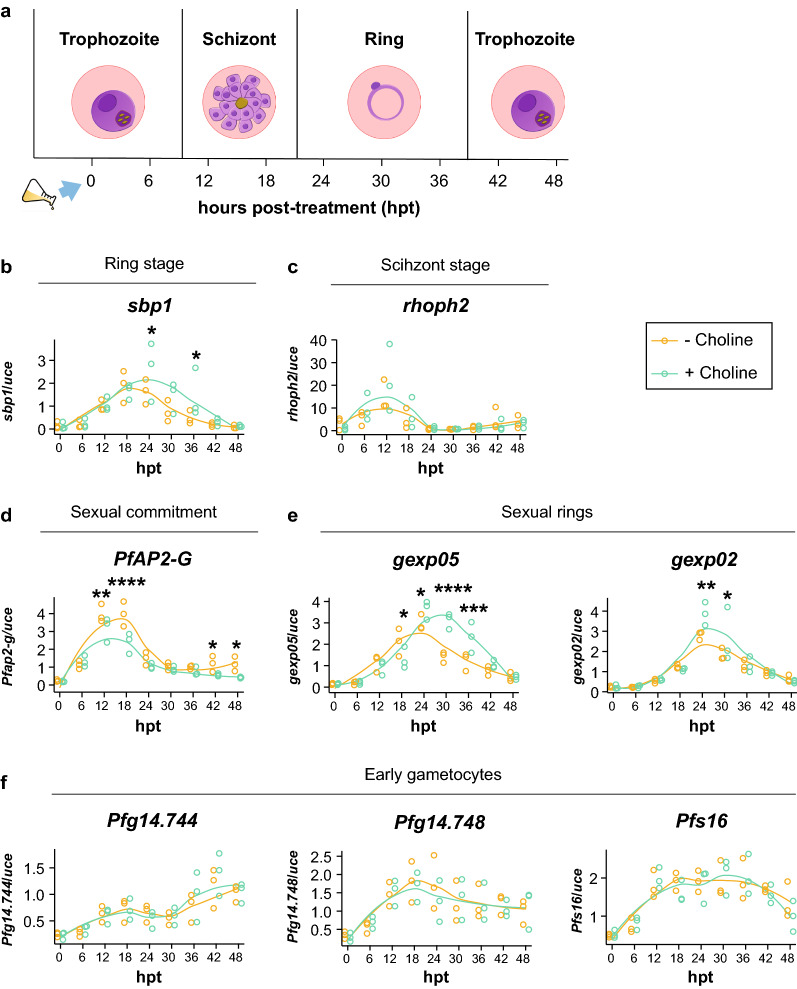
Choline represses *PfAP2-G* expression early in gametocytogenesis commitment. **a** Schematic illustration of the experimental setup: the transcriptional profiles of nine GRG markers were built for synchronized *NF54-gexp02-tdTomato* trophozoite-stage parasites treated under conditions of choline removal (- Choline) or choline supplementation (+ Choline). The flask and the arrow represent treatment induction (± Choline) starting at the first time point (0 hpt). RNA was extracted by the robotic unit at 6, 12, 18, 24, 30, 36, 42 and 48 hpt for RT-qPCR expression analysis. In parallel, parasitemia, % *gexp02*^+^ iRBC (proxy for the percentage of sexually committed cells) and stage composition in the culture were monitored by flow cytometry and Giemsa-stained smears (Additional file 13: Fig. S10). The predominant stage in each time span is also presented in the illustration for comparison with the transcriptional profiles. **b–f** Temporal transcriptional profiles of the ring and schizont stage-specific markers *sbp1* (**b**), *rhoph2* (**c**), the sexual commitment marker *PfAP2-G* (**d**), the sexual ring markers *gexp05* and *gexp02* (**e**), and the early gametocyte markers *Pfg14.744, Pfg14.748* and *Pfs16* (**f**) at the selected time points within 48 h. Mean transcript levels were calculated using the relative standard curve method and normalized to the transcript levels of *uce* in panels **b–f**. The smoothened trend line in the data was generated using a LOESS (locally weighted scatterplot smoothing) function with a span of 0.75 for the three independent biological repeats (*n* = 3). Two-way ANOVAs with post-hoc tests were run using estimated marginal means, with the R package ‘emmeans’ for all genes. *p < 0.05, **p ≤ 0.01, ***p ≤ 0.001 and ****p ≤ 0.0001

Using our robotic platform, we generated a high-resolution expression profile for nine *Pf* genes over the course of approximately one intraerythrocytic cycle (~ 48 h) over nine time points: 0, 6, 12, 18, 24 30, 36, 42 and 48 h post-choline removal (Fig. 4a). Trophozoite-stage parasites routinely cultured with 2 mM choline supplementation for a minimum of 2 weeks were washed, and culture media were replaced with fresh media containing either no choline (− Choline) to induce gametocytogenesis [19, 35] or 2 mM choline (+ Choline), which served as a control to maintain gametocytogenesis repression. *NF54-gexp02-Tom* parasites were sampled every 6 h over the course of 48 h (Fig. 4a). RNA was extracted by means of the robotic apparatus, and gene expression levels were evaluated using RT-qPCR analysis. This is, to the best of our knowledge, the first high-throughput RT-qPCR study to robustly monitor the gene expression profiles of *Pf* sexual markers at multiple time points post-induction of choline depletion.

The *Pf* genes analyzed by this wide RT-qPCR screening included: the sexual master regulator *PfAP2-G*, the sexual ring marker *gexp05,* the early gametocyte markers *Pfs16, Pfg14.744* and *Pfg14.748*, and two stage-specific genetic markers *sbp1* [6] and *rhoph2* [63], asexual rings and schizont-specific markers, respectively (Fig. 4b–f). As the *NF54-gexp02-Tom* line was used, we also decided to monitor the RNA expression patterns of the sexual ring (and early gametocyte) marker *gexp02* [19, 79]. Thus, its RNA levels could be complemented by detection of the proportion of *gexp02*^+^ parasites as a proxy for sexually committed parasites using flow cytometry (Additional file 13: Fig. S10b).

This extensive screen was performed in parallel with sampling the parasite culture at each time point using Giemsa-stained smears to (i) measure parasitemia levels and (ii) analyze blood stage composition (rings vs. trophozoites vs. schizonts) (Additional file 13: Fig. S10a-c).

We found that *PfAP2-G* presented a “wave-like” transcriptional profile, with peak expression at ~ 12–18 hpt (Fig. 4d) in the presence or absence of choline. Depletion of choline (- Choline) led to statistically significant upregulation of *PfAP2-G*, but only for a short interval between 12–18 hpt. During this time, most of the *Pf*-iRBCs are predominantly in the late schizont and early ring stage, with relatively low levels of trophozoites (Additional file 13: Fig. S10c). Then, as the parasites grow and transition into the later ring and trophozoite stages, the difference in *PfAP2-G* expression levels decreases, but becomes significantly upregulated again under choline depletion at the last time points (between 42–48 hpt). Importantly, the stage composition analysis revealed no significant changes in the proportion of schizonts or other stages at 18 h post-treatment between the samples (Additional file 13: Fig. S10c). The specific schizont marker, *rhoph2* [63], did not express differently between the treatments at any time points (Fig. 4c).

Overall, these data suggest that the changes observed in *PfAP2-G* expression were most probably due to ‘true’ alterations in transcriptional activity within the late schizonts/early rings at 12–18 hpt when choline is removed rather than due to differences in *Pf* stage composition. Choline depletion did not cause significant effects on parasite growth dynamics at these early time points.

In contrast to *PfAP2-G*, downstream GRGs were not affected by choline depletion as would have been expected (Fig. 4f). RNA expression levels of *Pfg14.744*, *Pfg14.748* and *Pfs16* were not affected by choline depletion over the course of 48 hpt (Fig. 4f). This could reflect the fact that the expression of these genes may be directly or indirectly induced by *PfAP2-G* or its downstream effectors later, after the initial 48 h of gametocytogenesis induction. These findings are consistent with previous data demonstrating that the expression of these early gametocyte genes usually occurs following the first 48 h of gametocytogenesis commitment [19]. Indeed, these markers were reported to be consistently expressed only once the committed parasites transition to stage I-II gametocytes [14, 17, 52] beyond the immediate 48 h of our analysis.

We identified a different transcriptional pattern for *gexp02* and *gexp05* (Fig. 4e), mostly considered as markers of the sexual ring stage and early gametocytes [16, 19, 79]. The expression levels of both genes peaked when most of the culture was in the ring-stage (24–36 hpt) as revealed by the stage composition analysis (Additional file 13: Fig. S10c) and by the temporal expression pattern of the ring-stage marker *sbp1* (Fig. 4b). Interestingly, for both *gexp02* and *gexp05*, transcript abundance was significantly higher in the presence of choline (Fig. 4e). This result is surprising as choline is expected to reduce sexual commitment levels [19, 33]. These data led us to suspect that choline treatment could also cause changes in parasite growth dynamics and thus stage composition, which could lead to changes in gene expression of these markers.

Evaluation of *Pf* growth in the presence of choline demonstrated a significant increase in parasitemia levels (Additional file 13: Fig. S10a). Moreover, stage composition analysis demonstrated a significantly increased proportion of ring-stage parasites at 30–36 hpt (Additional file 13: Fig. S10c) when most schizonts had already ruptured, when compared to the choline depleted samples. These findings were further supported by the detection of higher transcript abundance of the specific ring-stage marker *sbp1* at 24 and 36 hpt, in the choline-supplemented parasites, compared to the control (Fig. 4b). Taken together, these observations indicate a higher abundance of rings in the choline-supplemented culture compared to cultures lacking choline, suggesting that choline significantly affects *Pf* proliferation.

To confirm these findings, we quantified the numbers of daughter merozoites per schizont-stage parasite in each condition. Remarkably, we observed an average of ~ 19 merozoites/schizont under choline supplementation vs. ~ 15 merozoites/schizont when choline was removed (Additional file 13: Fig. S10d-e). Our data determined a significantly smaller number of daughter cells per schizont in parasites grown post-choline depletion, in alignment with previous studies reporting increased asexual proliferation and merozoite productivity in choline supplementation or Kennedy pathway activation [33, 34, 39, 80].

Taken together, we conclude that the observed increase in *gexp02* and *gexp05* transcript levels in the choline-treated samples may be explained by the positive effect of choline on parasite proliferation and merozoite productivity. This could have significantly increased the proportion of all, asexual and sexual, ring-stage parasites, thus enriching the number of copies of both asexual and sexual ring-stage RNA species in choline-treated parasites.

Furthermore, our results demonstrate a ‘true’ upregulation in *PfAP2-G* by means of choline depletion, which can be clearly observed at 12–18 hpt, in the late schizont and early ring stages. Downstream target genes may not change immediately after *PfAP2-G* induction and their expression levels may be biased, affected by variations in asexual stage composition. These data emphasize a need for caution when interpreting immediate changes in GRG expression rather than relying exclusively on RT-qPCR assays.

### The metabolites lactate and kynurenic acid do not immediately alter early GRG expression

We aimed to use our technology to explore the involvement of lactate and kynurenic acid in gametocytogenesis regulation. Both of these metabolites have been found to be abundant in severe malaria patients [45, 46, 58, 59, 81]. Indeed, kynurenic acid was found to accumulate in the cerebrospinal fluid of cerebral malaria patients [45, 46], and high levels of lactic acid have been detected in the circulation of patients suffering from severe malaria acidosis [58, 59, 81]. The latter was suggested as a potential gametocytogenesis regulator that does not affect parasite growth [43], although no direct *PfAP2-G* upregulation could be observed using RT-qPCR analysis. We therefore set out to evaluate whether lactate or kynurenic acid might alter immediate GRG expression and be involved in their regulation.

Based on our previous RT-qPCR results on GRG regulation using choline (Fig. 4), we directly tested the effect of lactate and kynurenic acid in trophozoites once the culture reached the late schizont stage (~ 16 hpt). NF54 wild-type *Pf* trophozoite-stage parasites were cultured in the presence or absence of choline (± Choline) and the two metabolites. The parasites were treated for 16 h with lactate or kynurenic acid at concentrations that mimicked the physiological levels estimated in severe malaria patients [45, 46, 58, 59]. *NF54-gexp02-Tom* parasites cultured in ± choline condition were used as a control, proven their ability respond to choline depletion during the previous screening (Fig. 4). The expression levels of the early gametocyte markers, *PfAP2-G* and *gexp02*, the ring stage marker *sbp1* and the schizont stage marker *rhoph2* were evaluated at the late schizont stage (~ 16 hpt) time point.

**Fig. 5:**
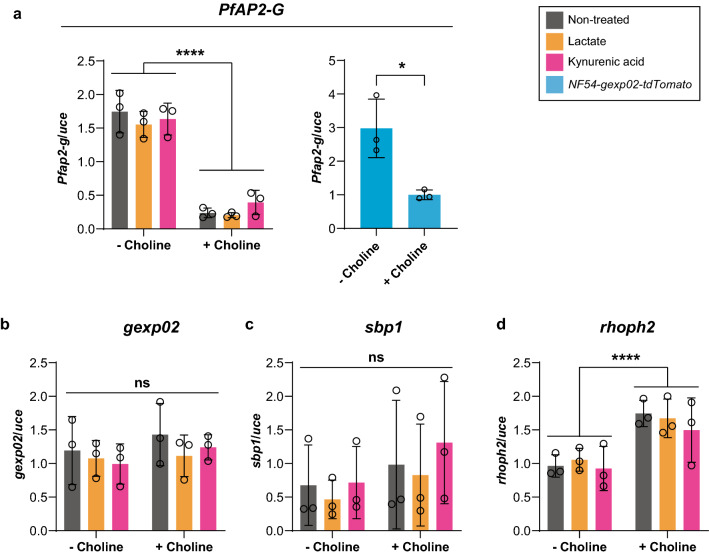
Severe malaria metabolites do not immediately alter gametocytogenesis regulation. Gene expression analysis during the late schizont stage for the gametocytogenesis master regulators *PfAP2-G* (**a**), *gexp02* (**b**)*, sbp1* (**c**) and *rhoph2* (**d**). In the case of *PfAP2-G*, the *NF54-gexp02-tdTomato* line was used as a control to illustrate choline treatment success (**a**, right panel). NF54 *Pf* parasites were cultured under choline removal (− Choline) or supplementation (+ Choline) conditions and exposed to lactate 5 mM or kynurenic acid 250 nM for 16 h. RNA was extracted at this time point only and gene expression analyzed by robot-automatized RT-qPCR. Data were normalized to *uce*. Error bars represent SD. Two-way ANOVA with post-hoc tests were run using estimated marginal means with the R package 'emmeans.' The significance presented in the figure corresponds to the two-way ANOVA for the “choline presence” factor. All the individual post-hoc contrasts were not significant, and not shown. ns = *p* ≥ 0.05 (non-significant), *p < 0.05 and ****p ≤ 0.0001

As shown in Fig. 5, indeed the NF54 parental line demonstrated choline-mediated *PfAP2-G* repression in the schizont stage (Fig. 5a, left), as was also observed for the *NF54-gexp02-Tom* line (Fig. 5a right). Nevertheless, at this time point neither of the two metabolites caused a significant change in *PfAP2-G* or *gexp02* expression (Fig. 5a–b). Of note, an elevation in the expression level of *rhoph2* under choline supplementation was observed under all treatments (Fig. 5d). *rhoph2* is a known marker for rhoptry biogenesis, a main component of the apical complex of merozoites [82]; therefore, the increase in its transcript abundance is likely the result of a choline-mediated increase of daughter cell productivity during schizogony (Additional file 13: Fig. S10d).

These data suggest that treatment of trophozoites with kynurenic or lactic acids does not immediately affect *Pf* sexual commitment. Overall, this study lays the groundwork for future studies that employ robot-automated systems like the one developed here to evaluate the expression profiles of multiple GRG markers at different time points and under multiple conditions to advance our understanding of this essential process in malaria transmission.

## Discussion

**Fig. 6:**
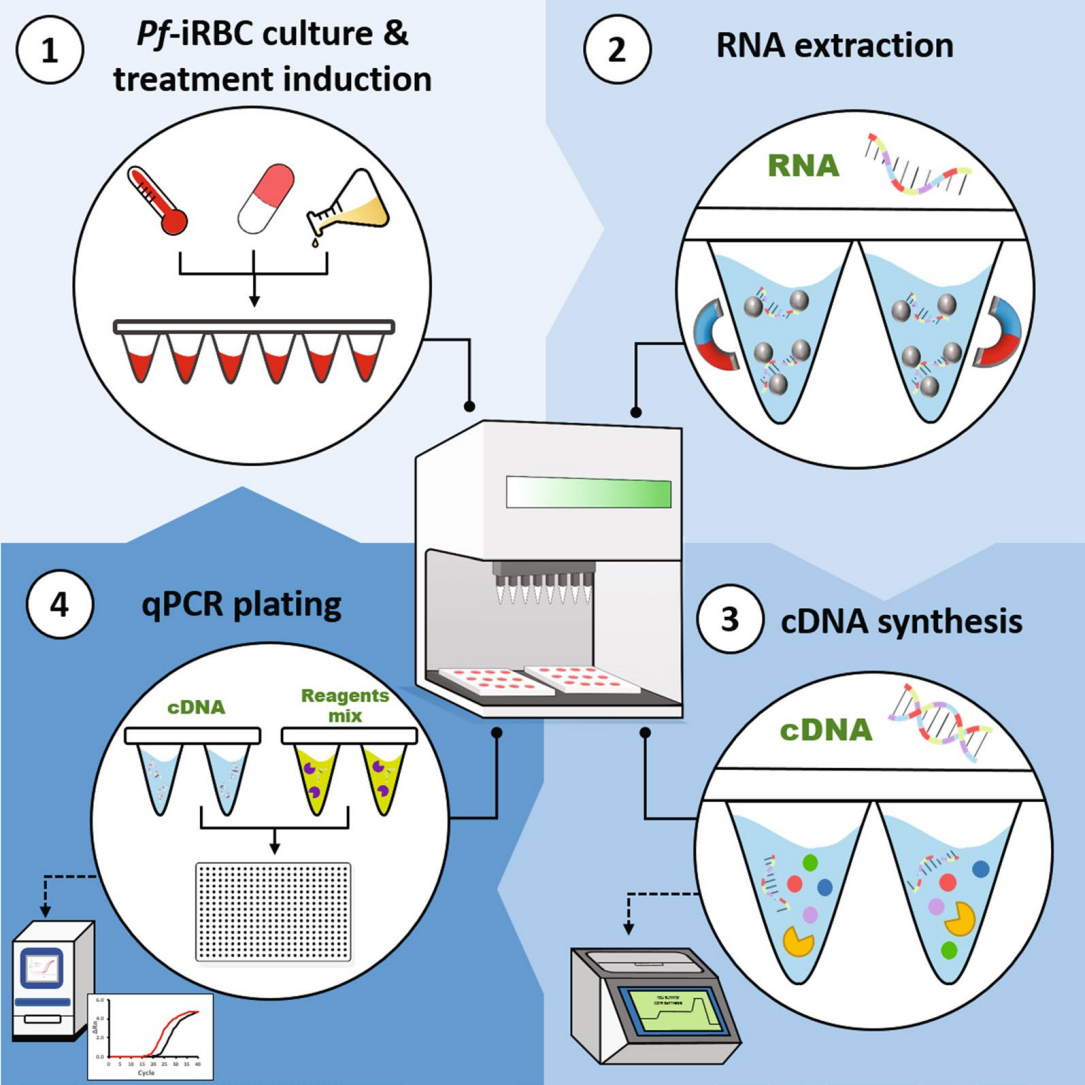
Schematic illustration of the robot-automated RT-qPCR platform for monitoring *Pf* gene expression profiles. The goal of the automated RT-qPCR platform is to simultaneously evaluate multiple conditions that may affect gene expression regulation. First, *Pf*-iRBCs are cultured under different treatments induced by robot-automated culturing (work in progress), incubation and Giemsa smear monitoring (Step 1). RNA is then extracted from *Pf*-iRBCs by a completely automated high-throughput method, up to 96 samples simultaneously (Step 2). cDNA is next prepared from up to 96 samples simultaneously by robot-automated RNA dilution and reverse-transcription master mix aliquoting (Step 3). Finally, 384-well RT-qPCR plates are prepared using the robotic system by aliquoting the cDNA solutions and the RT-qPCR reaction mix (Step 4)

Gametocytogenesis is a fundamental step in the life cycle of the malaria parasite; however, our knowledge of the factors underlying this key differentiation process remains highly elusive. Here, we developed a RT-qPCR automated robotic system, composed of three hands-free sequential steps involving the harvesting of RNA, cDNA synthesis and RT-qPCR plating (Fig. 6). Our technology enabled us to robustly monitor mRNA expression profiles for a large panel of GRGs from small-volume samples of *Pf*-iRBCs. By this, this advanced method opens up new possibilities for extensively studying the molecular mechanisms underlying gametocytogenesis and investigate potential regulators. Even though in the present study we used only some genes of the panel to evaluate early sexual commitment, this large panel can aid in evaluating gametocytogenesis regulation in multiple parts of this process and can be applied to study a wide variety of processes in *Pf* transcriptional regulation apart from sexual form development.

We demonstrated the robustness of our method to harvest total RNA from small-volume samples of *Pf*-iRBCs (Fig. 1, Additional file 4: Fig. S1, Additional file 5: Fig. S2, Additional file 6. Fig. S3), a key advantage of our system compared to other methods [63, 83]. This advantage enables higher sample processing capacity. We have shown the RNA yield to be of high quality as it produces reliable standard curves when testing gene expression profiles (Fig. 2, Additional file 2: Table S2 and Additional file 3: Table S3, Additional file 7, Fig. S4).

The use of this platform enabled us to gain new insights into the necessary elements required when studying immediate GRG expression responses. In particular, we explored the effects of two regulators, DHA treatment (Fig. 3) and choline depletion (Fig. 4). While we could detect significant changes in the expression of the sexual commitment marker *PfAP2-G*, both treatments also resulted in strong changes in parasite growth and stage composition when compared to the non-treated control (Additional file 10: Fig. S7 and Additional file 13: Fig. S10). A short 3-h exposure to DHA resulted in a higher proportion of trophozoites and schizonts at 25–30 hpt, most likely due to DHA toxicity, while choline supplementation yielded a larger proportion of ring-stage parasites 30–36 hpt. These variations in stage composition occurred in parallel to the observed changes in GRG as detected by RT-qPCR, highlighting the need to incorporate asexual markers to the RT-qPCR analysis (ideally markers of ring, trophozoite and schizont stages; Figs. 3, 4).

**Fig. 7:**
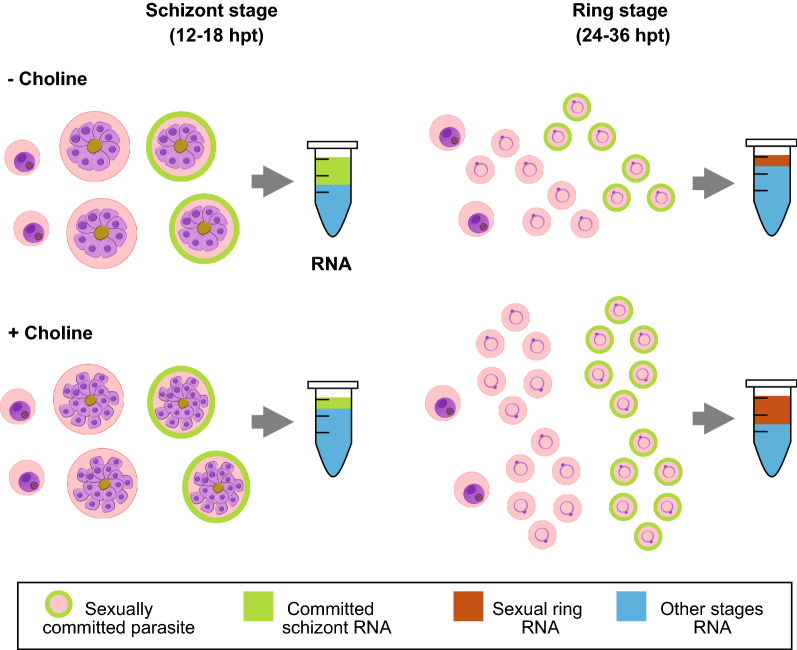
Proposed model of the mixed effects of choline on GRG expression and growth. In choline-depleted parasites (upper panel), a certain percentage of asexual parasites commit to sexual differentiation (green lines) and express higher levels of *PfAP2-G*, mainly at the late schizont stage (12–18 hpt), compared to choline-supplemented parasites (lower panel), in which the expression of *PfAP2-G* is effectively repressed. This was correspondingly observed in the RT-qPCR assay performed over the extracted RNA at this stage (Fig. 4b). After schizont rupture at 24–36 hpt, the sexual rings (green lines) derived from these *PfAP2-G*^+^ committed schizonts were initially expected to express their specific GRGs (*gexp02* and *gexp05*), as if choline would affect *PfAP2-G* expression only. However, choline supplementation increases merozoite productivity during schizogony (represented by schizonts with a higher number of daughter merozoites in the diagram), yielding a significantly higher level of parasitemia and a significantly higher proportion of ring-stage parasites in the culture at 24–36 hpt (Additional file 13: Fig. S10). Since bulk techniques such as RT-qPCR analyze the differences in transcript abundance of the total parasitic population at a given time point, RNA extracted from the pool of cells is an overall mixture of the proportional contributions of each one of the developmental stages present in the sample. Assuming choline increases merozoite productivity equally in asexual and sexual parasites, the extracted RNA at 24–36 hpt had a much higher proportion of both sexual and asexual ring RNA in the choline-supplemented samples. Thus, RT-qPCR evaluation of GRG expression 24–36 hpt demonstrated a higher abundance of GRG transcripts under conditions of choline supplementation. The choline-mediated % *gexp02*^+^ iRBC reduction was not presented in this model, as it was only found to be significant after 48 hpt (Additional file 13: Fig. S10b)

These data lead us to hypothesize three potential scenarios of how these treatments may affect GRG transcript abundance within the initial 48 h course of treatment: (i) the treatments directly cause a true change in GRG expression regulation; (ii) the treatments result in a change in stage composition; as a consequence, some GRGs that are tightly linked to a specific stage become more or less abundant (Fig. 7); or (iii) the treatments yield a combined effect, leading to a change in stage composition, as well as to a true change in GRG transcriptional regulation. Moreover, analyzing the results of a mixed culture could lead to biased conclusions, since not all stages of development produce the same amount of RNA [84, 85]. This could potentially lead to a situation in which the relative abundance of a target gene may be diluted by “RNA contamination” from other stages.

We therefore suggest that the observed shift in the expression pattern of *PfAP2-G* by DHA treatment in gametocytogenesis-inducing conditions (absence of choline) (Fig. 3b) could at least partially result from the asexual stage composition alteration, since DHA did not significantly increase the gametocyte abundance (Additional file 11: Fig. S8). The presence of a higher number of schizonts (which express high levels of *PfAP2-G*) due to growth arrest by this antimalarial drug could significantly contribute to the delayed peak in the *PfAP2-G* expression (Scenario ii). This finding could also explain the discrepancies in the effects of DHA on *PfAP2-G* expression reported in previous studies [35, 40], as it seems like the effect of DHA in gametocytogenesis considerably varies according to the experimental setup in gametocyte-inducing or repressive conditions by choline supplementation/Kennedy pathway activation. Additional studies using single-cell transcriptional profiles could help in determining the possible direct induction of *PfAP2-G* expression and gametocyte abundance by DHA and other antimalarial drugs in the absence of choline-mediated gametocyte repression.

In the case of choline depletion, we observed at least two possible GRG alteration mechanisms. First, since we could not detect a difference in stage composition between the treatments during 12–18 hpt, we conclude that the detected upregulation of *PfAP2-G* (Fig. 4d) was due to the direct influence of choline depletion on its transcriptional regulation (Scenario i). In fact, the effect of choline depletion was also propagated in the next parasite generation, 42–48 hpt. This finding aligns with previous studies describing choline as a gametocytogenesis repressor [19, 33, 35].

In contrast, the unexpected changes in expression of the sexual ring markers *gexp02* and *gexp05* (30–36 hpt) could be interpreted by the differences in stage composition attributed to choline (Scenario ii), given that *gexp02* and *gexp05* are mainly expressed during the ring phase (Fig. 4e). The abundance of these two gene products is regulated mainly by two factors: (i) the proportion of ring-stage parasites in the *Pf* culture and (ii) the sexual conversion rate (%GCR). In the current study, our data support the notion of an increased proportion of total ring-stage iRBCs during 30–36 hpt, but not a significant change in the percentage of *gexp02*^+^ sexually committed cells at this time point (Additional file 10: Fig. S7). In our study, the percentage of *gexp02*^+^ sexually committed cells in the *NF54-gexp02-Tom* reporter line was significantly different between choline depletion and supplementation only after 48 h post-induction compared to a previous study where a significantly higher proportion of *gexp02*^+^ sexually committed parasites was already observed in the sexual ring stage after reinvasion of induced trophozoite-stage parasites (after 10–15 hpi) [19].

Thus, we suggest that the main driving force behind the increased abundance of *gexp02* and *gexp05* observed at 30–36 hpt, when most of the parasites are in the ring-stage post reinvasion, lies in the difference in stage composition (illustrated in Fig. 7) due to increased merozoite production by choline supplementation observed here and in parasites with an active and functional Kennedy pathway in a previous study [80]. Single-cell technologies can aid in clarifying the complex roles of choline in GRG regulation as this metabolite causes a true change in *PfAP2-G* regulation (Scenario i) but may also exert a mixed effect on the expression of some of its targets (Scenario iii), depending on the timing of the RT-qPCR analysis.

Considering these results, we primarily conclude that different treatment conditions may also cause changes in asexual growth and/or stage transition additional to their potential role in regulating gametocytogenesis, which may complicate the interpretation of GRG expression analysis in early time points. Accordingly, we recommend that these assays should be conducted at the appropriate time frame: namely, before the treatment causes a significant difference in these parameters, similar to treatment with lactate and kynurenic acid (Fig. 5), which did not affect parasite growth at the measured time point. This consideration is important for many gametocytogenesis-regulating candidates such as antimalarial drugs, since it was shown that a generalized growth stress response is linked to gametocyte development [40], which can impact both asexual and sexual parasite populations to different degrees.

Importantly, asexual stage composition analyses must be performed in parallel to gene expression evaluation by RT-qPCR to better understand the combined effects of treatments in sexual and asexual parasite populations. For example, we found that the preferred hours for *PfAP2-G* expression analysis are between ~ 12–18 hpt, when the parasites are mostly in the late schizont/early ring stage, and *PfAP2-G* reaches peak expression [21]. For increased accuracy, we suggest a time course analysis using an automated method such as that presented here, which greatly aids in efficient analysis of large numbers of samples. These conclusions are relevant to any bulk gene expression analysis methods such as RT-qPCR and RNA-seq.

Our data additionally showed that, despite the upregulation of *PfAP2-G*, there was no significant change in its downstream GRG targets (*gexp05, Pfg14.744*, *Pfg14.748* and *Pfs16*) within the 48-h time period evaluated. In alignment with our data, a previous study revealed that LysoPC depletion did not upregulate *PfAP2-G* target genes 16 h post-induction [33]. Interestingly, in other studies using various techniques to monitor the response of the target genes to *PfAP2-G* transgenic activation, it was possible to observe induction of these target genes [13, 20]. For instance, when evaluating the target transcriptome of *PfAP2-G* via transgenic lines that stabilize the *PfAP2-G* protein [13] or conditionally control its expression [20], significant upregulation of *PfAP2-G* targets (*Pfg14.744, Pfg14.748*, *Pfg27* and *Pfs16*) was detected within the 48 h time period. One possible explanation could be that artificial transgenic induction of *PfAP2-G* may lead to increased transcript abundance and a more rapid accumulation of *PfAP2-G* transcripts via its positive feedback loop as compared to choline depletion, as portrayed in this study, which may take longer than 48 h to stably induce the expression of the evaluated GRGs [13]. Hence, in the case of some candidate inducers, 48 h post-*PfAP2-G* induction may not allow sufficient time to detect immediate downstream GRG regulation.

The application of our developed method allowed us to gain a broader mechanistic insight into the regulation of some of the earliest gametocytogenesis genes and highlighted the importance of evaluating in parallel the changes in sexual gene expression with the potential effects of a treatment on the asexual stage transition instead of only one of them, which could lead to bias in the data interpretation. Since we observed that non-homogeneous stage distribution increases variability in gene expression and biases the analysis, we recommend to either focus the analysis on previous time points when the stage distribution is constant (for example, the case of *PfAP2-G* which peaks its expression 12–18 hpt in trophozoite-stage induction) or, for later gametocyte markers whose expression can be inconsistent during the first 48 h of analysis, it is recommended to analyze after 48 hpt to ensure that all the parasites induced into gametocytogenesis express them more stably.

## Conclusions

We developed a fully automated high-throughput RT-qPCR method that provides a significant improvement in studying the effect of treatments on *Pf* gene regulation, focusing on gametocytogenesis. This system has the potential to also benefit the malaria clinical field, as it can be alternatively applied in the quantification of gametocytemia in clinical samples from patients in high throughput from minimal blood sample quantities. Since the gene panel spans through multiple stages of the sexual differentiation process, this platform could also be potentially useful to quantify specific gametocyte stages in patients, detect asymptomatic carriers and study the roles of treatments and environmental factors in the parasite sexual development in the field. In addition, this work emphasizes the importance of parameters necessary to evaluate in parallel when studying the effect of external factors in sexual commitment of malaria parasites via alterations of mRNA levels when evaluated by RT-qPCR. The importance of stage composition was thoroughly demonstrated in previous studies using transcriptional evaluation via microarrays, RNA-seq and single-cell transcriptomics [76, 86, 87]. Nevertheless, we here demonstrate the importance of evaluating the impact of treatments on the stage composition and asexual cycle dynamics when studying gene expression and gametocytogenesis commitment by RT-qPCR, which has been widely overlooked in previous studies that use RT-qPCR as the main readout technique. Future research is needed to understand, in greater depth, the complex signaling required for gametocytogenesis completion. Automated pipelines would substantially aid in meticulously elucidating the process.

## Supplementary Information

**Additional file 1: Table S1.** Primers used in this study. The melting temperature (Tm) and amplicon size were predicted using the primer-BLAST (NCBI) web portal. An alignment analysis was also carried out in primer-BLAST against all the non-redundant nucleotide databases. No off-targets were found, except for those to be expected in *Plasmodium* for each gene. The ID number presented for each gene corresponds to the *Pf* 3D7 strain.

**Additional file 2: Table S2.** Representative standard curve statistical parameters for each gene in the early gametocytogenesis panel. The statistical parameters of correlation coefficient (r^2^), slope and y-axis intersection were obtained from the QuantStudio Real-Time Software (Applied Biosystems) and efficiency calculated as reported [63]. Data represent one exemplary biological repeat.

**Additional file 3: Table S3.** Mean standard curve statistical parameters for each gene in the early gametocytogenesis panel. Data represent the mean of three independent biological replicates of each standard curve and correspond to the curves shown in the Additional file 7: Fig. S4. Statistical parameters were derived from the mixed model ANOVA for batch effect applied to the three independent repeats. The r^2^ parameter does not represent the correlation between quantity and Ct, but among the three repeats and the best-fitting line defined by the statistical model (as a measure of reproducibility) and further deviates from ideality (*r*^*2*^ = 1.0) because of biological variations between repeats (batch effect).

**Additional file 4: Figure S1.** Spectrophotometric determination of the mean A_260_/A_230_ purity ratios of the RNA extracted from *Pf*-iRBC trophozite- (**a**) or ring-stage (**b**) cultures using (i) robot-automated magnetic binding (rMB), (ii) solid-phase extraction (SPE) or (iii) liquid–liquid extraction (LLE) methods presented in Fig. 1. Panels **a**, **b** present the mean of three biological replicates (*n* = 3) with three technical replicates each. Error bars represent SD. A one-way ANOVA with Tukey’s multiple comparisons test was performed for panels **a**–**b**. ns = *p* ≥ 0.05 (non-significant), **p ≤ 0.01 and ****p ≤ 0.0001.

**Additional file 5: Figure S2.** Agarose gel electrophoresis for RNA samples extracted using either the robot-automated magnetic binding (rMB), solid phase extraction (SPE) or liquid–liquid extraction (LLE) methods. The 28S and 18S rRNA bands are indicated in the right border, and the size marker (SM) is presented in the first lane. An example of one representative biological repeat out of three performed is presented.

**Additional file 6: Figure S3.** Capillary electropherograms for one representative sample of each biological replicate. Importantly, all of the LLE samples presented RNA concentrations much lower than the rMB and SPE methods in the quantification provided by capillary electrophoresis (data not shown). This suggests that the RNA quantification based on spectrophotometry at 260 nm (Fig. 1a, b) for the LLE samples largely overestimates its actual concentration, probably because of a large degree of protein contamination. Proteins also absorb at 260 nm and can interfere with RNA quantification when present in large quantities [68, 88] as revealed in the A_260_/A_280_ purity ratio for this method.

**Additional file 7: Figure S4.** Mean standard curves for the GRG panel in ring and trophozoite stage parasites in three independent biological repeats (*n* = 3). Standard curves were entirely generated by means of the robotic system, by a serial 1:5 dilution of a standard pool of ring- or trophozoite-derived cDNA. The statistical parameters of one representative example of each biological repeat, used for actual relative quantification, are presented in Additional file 2: Table S2. The statistical correlation and efficiency parameters of the average of the three repeats in a mixed model ANOVA for batch effect were used to evaluate reproducibility of the standard curves, and are presented in Additional file 3: Table S3. Data represent the mean of three biological replicates (*n* = 3) with three technical replicates each. Error bars represent SD.

**Additional file 8: Figure S5.** Representative melting curves of the early gametocytogenesis gene panel applied to ring and trophozoite stage parasites. The derivative of the reporter dye fluorescence (dF) against temperature is presented.

**Additional file 9: Figure S6.** No reverse transcription RT-qPCR assay. RNA extracted from trophozoite-stage parasites was subjected to reverse transcription to generate cDNA (RT-treated) or directly used as input (Non RT-treated) for the RT-qPCR reaction, using a no-template control as a control for no amplification. Amplification was considered negligible if the Ct value was above the low amplification threshold of Ct = 35 [63] (represented as a horizontal line in the plot). For those repeats where amplification was negative (Ct ≥ 40), the Ct value was considered equal to 40.0. The symbol ♦ represents those bars where at least one of the biological repeats did not present any amplification and was thus considered *Ct* = 40. Data represent the mean of three biological replicates (*n* = 3) with three technical replicates each. Error bars represent SD.

**Additional file 10: Figure S7.** Growth dynamics and stage composition analysis during DHA treatment. **a** Parasitemia quantified by Giemsa-stained smears for the growth assay presented in Fig. 3H. Parasitemia was determined by manual counting of *Pf*-iRBC percentage relative to the total RBC in least 10 fields, in three technical replicates of each one of the three independent biological replicates. **b** Stage composition analysis of the Giemsa-stained smears of the growth assay after 24, 48 or 72 hpt. Mean percentages of ring, trophozoite and schizont stage parasites were calculated in at least 50 parasites over 10 fields in three technical replicates of each one of the three independent biological replicates. Error bars represent SD. Two-way ANOVA with post-hoc tests were run using estimated marginal means with the R package ‘emmeans’ for Panel **a**, and one-way ANOVA with Dunnett’s multiple comparisons test was performed for each stage in each time point independently in Panel **b**. * = *p* < 0.05 and ** = *p* ≤ 0.01.

**Additional file 11: Figure S8.** Sexual assay to measure *Pf* sexual conversion. *NF54* trophozoite-stage parasites were treated with DHA 10 nM, DMSO 0.1% (solvent control) or non-treated. Media were replaced daily for 7 days supplemented with 10% human serum and NAG. Asexual % parasitemia and % gametocytemia were determined manually on day 1 and day 7, respectively, using Giemsa-stained blood smear counting over 10 fields. Percentage of sexual conversion was calculated by dividing the day 7% gametocytemia by the day 1% asexual parasitemia and multiplying by a factor of 100%. Data represent the mean of three independent biological repeats, the error bars represent SD, and one-way ANOVA with Dunnett’s multiple comparisons test was performed. ns = *p* > 0.05.

**Additional file 12: Figure S9.** Early gametocytogenesis gene expression screening with chloroquine. **a** Mean gene expression of *PfAP2-G, gexp05, Pfg14.744 Pfg14.748, Pfs16* and *Pfs25* of ring-stage *Pf* cultures exposed to 40 nM choloroquine (CQ) for 24 h or non-treated (NT); data were normalized to *uce*. **b** Mean gene expression of the parasitic genes *PfAP2-G, Pfg14.744* and *Pfs25* of trophozoite-stage cultures exposed to increasing concentrations of CQ over the course of 4 h or non-treated (NT); data were normalized to *uce*. Data represent the mean of three independent biological repeats. Error bars represent SD. One-way ANOVA with Dunnett’s multiple comparisons test was performed for Panel **b**, and an unpaired t-test was performed for Panel **a**. *P*-values were adjusted for batch effect in Panel **a**. ns represents *p* ≥ 0.05 (non-significant).

**Additional file 13: Figure S10.** Growth dynamics and stage composition analysis during choline treatment. **a** Parasitemia and **b** Percentage of *gexp02*^+^ iRBC quantified by flow cytometry for the experiment presented in Fig. 4. Parasitemia was determined by the percentage of gated Hoechst-33342 positive *Pf*-infected cells and %*gexp02*^+^ as the percentage of gated Hoechst-33342 and tdTomato^+^ double-positive cells, as explained in the Methods section. **c** Stage composition analysis of the Giemsa-stained smears obtained in each one of the time points evaluated, as illustrated in Fig. 4. Mean percentages of ring, trophozoite and schizont stage parasites were calculated in at least 50 parasites over 10 fields in three technical replicates of each one of the three independent biological replicates. **d** Mean number of merozoite daughter cells per schizont, quantified at 12 hpt in the choline-depleted and -supplemented cultures (Fig. 4). Mean merozoites/schizonts were quantified in at least 10 representative schizonts in each one of the three biological replicates per treatment. **e** Representative microscopic images of schizonts of choline-depleted and -supplemented cultures in Giemsa-stained smears. Error bars represent SD. Two-way ANOVA with post-hoc tests were run using estimated marginal means with the R package ‘emmeans’ (Panels **a** and **b**). A one-way ANOVA with Dunnett’s multiple comparisons test was performed for each stage in each time point independently (Panel **a**), and an unpaired *t*test was performed (Panel **d**).*p < 0.05 and **p ≤ 0.01.

**Additional file 14:** Figure S11. Population scatter plots and flow cytometry gating strategy for the DHA growth assay. NF54 *Pf*-iRBCs were treated as described in Fig. 3. Cells were harvested and stained with Hoechst-33342 and thiazole orange. Initially, a viable singlet RBC population (R1) was gated in the forward scatter vs. side scatter plot (not shown) to exclude doublets and cell debris. The singlet infected RBC population (R2) gating strategy is presented in this figure, with the thiazole orange (TO) log_2_ fluorescence intensity in the x-axis and the Hoechst-33342 log_2_ fluorescence intensity in the y-axis of each scatter plot. The population of *Pf*-iRBCs was considered to be those cells positive for the DNA stain Hoechst 33342 (gated in red) and the RNA stain. TO staining served as a control for non-specific staining (TO^+^ only, non-gated). Parasitemia was calculated as the percentage of *Pf*-iRBCs from the total initial singlet RBC population (uninfected RBCs). Analysis was performed using a ZE5^™^ flow cytometer (Bio-Rad). This figure presents representative scatter plots from one technical repeat out of three performed per biological repeat.

## Data Availability

The datasets generated during the current study are available in the public repository Zenodo, https://doi.org/10.5281/zenodo.6323532, https://doi.org/10.5281/zenodo.6323532.

## Abbreviations

*Pf*: *Plasmodium falciparum*
RT-qPCR: Quantitative polymerase chain reaction
GRG: Gametocytogenesis-related gene
GCR: Gametocyte commitment rate
