# Supplementary material for: High-throughput analysis of the transcriptional patterns of sexual genes in malaria
Source: Parasit Vectors. 2023 Jan 13;16:14. doi: 10.1186/s13071-022-05624-w (PMC9838061; doi:10.1186/s13071-022-05624-w)
Supplement: Supplementary file 1 — Additional file 1: Table S1. Primers used in this study. The melting temperature (Tm) and amplicon size were predicted using the primer-BLAST (NCBI) web portal. An alignment analysis was also carried out in primer-BLAST against all the non-redundant nucleotide databases. No off-targets were found, except for those to be expected in Plasmodium for each gene. The ID number presented for each gene corresponds to the Pf 3D7 strain. [file 13071_2022_5624_MOESM1_ESM.pdf]

Gene (ID)	Primer	Sequence	Length (nt)	Predicted Tm (°C)	Predicted amplicon size (bp)
pk4 (PF3D7_0628200)	Forward	5’-AACCCAGCTCCTCATGCTAC-3’	20	59.46	120
	Reverse	5’-CGTTTCGGTTCGAGAATTTGGT-3’	22	59.78	
uce (PF3D7_0812600)	Forward	5’-GGTGTTAGTGGCTCACCAATAGGA-3’	24	62.14	89
	Reverse	5’-GTACCACCTTCCCATGGAGTA-3’	21	58.53	
PfAP2-G (PF3D7_0935400)	Forward	5’-GATGGTGGGAAAATGGAAGACG-3’	22	59.58	79
	Reverse	5’-GCCATTCTCAATGCACCATCA-3’	21	59.25	
gexp05 (PF3D7_0936600)	Forward	5’-CGAGATTATCCCTTTTGGGGCT-3’	22	60.16	152
	Reverse	5’-TTCACCACTTCTCAAACAACCAC-3’	23	59.56	
Pfg14.744 (PF3D7_1477300)	Forward	5’-AGGATAGACACGCGCAACAAT-3’	21	60.41	98
	Reverse	5’-TGTGCCTGTTTATTCCTCGCA-3’	21	60.27	
Pfg14.748 (PF3D7_1477700)	Forward	5’-AATTCAAGGGTAGTTCCTAGAGCA-3’	24	59.22	80
	Reverse	5’-GCACTCGTAATTCTAACACTGGG-3’	23	59.38	
Pfs16 (PF3D7_0406200)	Forward	5’-GGTGCCTCTCTTCATGCTGT-3’	20	60.04	84
	Reverse	5’-AGGCATTTTGTCAGCAGAATCTT-3’	23	59.17	
Pfg27 (PF3D7_1302100)	Forward	5’-GGACGCAGCAGCAAGAATAAG-3’	21	59.94	81
	Reverse	5’-ACGGGTAAAGCAGGTATTGGC-3’	21	60.68	
Pfs25 (PF3D7_1031000)	Forward	5’- TCTTTTCCTTTTCATTCAACTTAGCA-3’	26	57.99	96
	Reverse	5’- CCACTCATCTGAATTAAAAATCCTCTT-3’	27	58.04	
sbp1 (PF3D7_0501300)	Forward	5’- GGCATCTGCAACTACCGAAT-3’	20	58.33	124
	Reverse	5’- GCTTGAAAAACCGTCATCGT-3’	20	57.32	
gexp02 (PF3D7_1102500)	Forward	5’-AAGTTTAGACGCTATGCCTGCT-3’	22	60.09	70
	Reverse	5’-ATGCAAAAGCTTCATTTTTGGATTC-3’	25	58.09	
rhoph2 (PF3D7_0929400)	Forward	5’-TGTTGCTGTCCATATTTAGTTTT-3’	23	54.79	127
	Reverse	5’-AATATATCGCTACATAACTTCGT-3’	23	53.34	
