# Supplementary material for: High-throughput analysis of the transcriptional patterns of sexual genes in malaria
Source: Parasit Vectors. 2023 Jan 13;16:14. doi: 10.1186/s13071-022-05624-w (PMC9838061; doi:10.1186/s13071-022-05624-w)
Supplement: Supplementary file 2 — Additional file 2: Table S2. Representative standard curve statistical parameters for each gene in the early gametocytogenesis panel. The statistical parameters of correlation coefficient (r2), slope and y-axis intersection were obtained from the QuantStudio Real-Time Software (Applied Biosystems) and efficiency calculated as reported [63]. Data represent one exemplary biological repeat. [file 13071_2022_5624_MOESM2_ESM.pdf]

Gene	Stage	% Efficiency = $(10^{-1/m} - 1) * 100$	r^2	y-intercept
pk4	Rings	93.419	0.995	17.695
	Trophozoites	92.05	0.991	18.381
uce	Rings	91.767	0.998	19.393
	Trophozoites	94.383	0.998	19.39
PfAP2-G	Rings	93.831	0.996	21.149
	Trophozoites	95.052	0.993	22.706
gexp05	Rings	92.834	0.996	15.621
	Trophozoites	94.671	0.995	20.02
Pfg14.744	Rings	99.959	0.996	20.638
	Trophozoites	94.682	0.995	18.994
Pfg14.748	Rings	100.766	0.994	22.618
	Trophozoites	99.375	0.997	20.575
Pfs16	Rings	97.852	0.998	16.528
	Trophozoites	97.72	0.997	17.189
Pfg27	Rings	94.368	0.997	17.756
	Trophozoites	99.78	0.998	15.646
Pfs25	Rings	95.074	0.995	22.74
	Trophozoites	90.256	0.99	23.294
sbp1	Rings	97.161	0.992	17.025
	Trophozoites	94.078	0.994	24.327
