# Supplementary material for: High-throughput analysis of the transcriptional patterns of sexual genes in malaria
Source: Parasit Vectors. 2023 Jan 13;16:14. doi: 10.1186/s13071-022-05624-w (PMC9838061; doi:10.1186/s13071-022-05624-w)
Supplement: Supplementary file 3 — Additional file 3: Table S3. Mean standard curve statistical parameters for each gene in the early gametocytogenesis panel. Data represent the mean of three independent biological replicates of each standard curve and correspond to the curves shown in the Additional file 7: Fig. S4. Statistical parameters were derived from the mixed model ANOVA for batch effect applied to the three independent repeats. The r2 parameter does not represent the correlation between quantity and Ct, but among the three repeats and the best-fitting line defined by the statistical model (as a measure of reproducibility) and further deviates from ideality (r2 = 1.0) because of biological variations between repeats (batch effect). [file 13071_2022_5624_MOESM3_ESM.pdf]

Gene	Stage	Mean % Efficiency = $(10^{-1/m} - 1) * 100$	Mean r^2	Mean y-intercept
pk4	Rings	92.38	0.99	17.40
	Trophozoites	91.75	0.99	18.69
uce	Rings	90.85	1.00	19.44
	Trophozoites	92.56	0.99	19.00
PfAP2-G	Rings	93.52	0.99	21.42
	Trophozoites	95.27	0.99	22.89
gexp05	Rings	91.76	1.00	15.51
	Trophozoites	93.39	0.97	20.52
Pfg14.744	Rings	97.87	0.99	20.46
	Trophozoites	92.29	0.99	19.21
Pfg14.748	Rings	93.84	0.95	21.68
	Trophozoites	96.00	1.00	20.54
Pfs16	Rings	96.08	1.00	16.41
	Trophozoites	97.22	0.99	16.88
Pfg27	Rings	91.80	0.99	17.25
	Trophozoites	96.40	0.98	15.87
Pfs25	Rings	90.75	0.99	22.30
	Trophozoites	85.40	0.96	23.63
sbp1	Rings	95.21	0.99	16.62
	Trophozoites	99.58	0.99	24.69
