# Supplementary figures and images for: High-throughput analysis of the transcriptional patterns of sexual genes in malaria
Source: Parasit Vectors. 2023 Jan 13;16:14. doi: 10.1186/s13071-022-05624-w (PMC9838061; doi:10.1186/s13071-022-05624-w)

RNA spectrophotometric  
purity ( $A_{260}/A_{230}$ )

**a**

Trophozoites

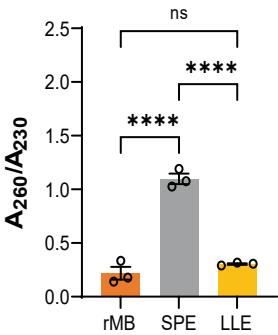

**b**

Rings

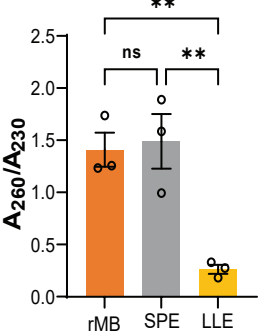

Supplement: Supplementary file 4 — Additional file 4: Figure S1. Spectrophotometric determination of the mean A260/A230 purity ratios of the RNA extracted from Pf-iRBC trophozite- (a) or ring-stage (b) cultures using (i) robot-automated magnetic binding (rMB), (ii) solid-phase extraction (SPE) or (iii) liquid–liquid extraction (LLE) methods presented in Fig. 1. Panels a, b present the mean of three biological replicates (n = 3) with three technical replicates each. Error bars represent SD. A one-way ANOVA with Tukey’s multiple comparisons test was performed for panels a–b. ns = p ≥ 0.05 (non-significant), **p ≤ 0.01 and ****p ≤ 0.0001. [file 13071_2022_5624_MOESM4_ESM.pdf]

Trophozoites

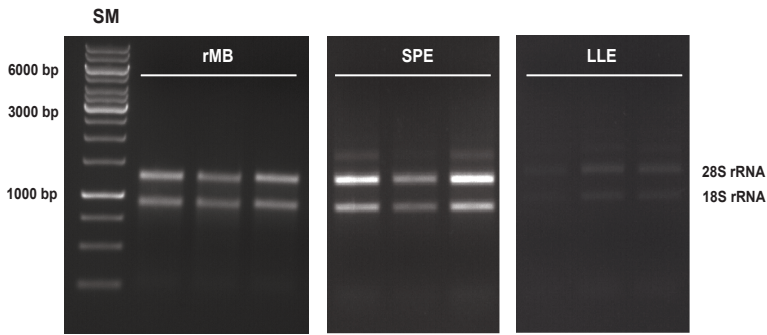

Rings

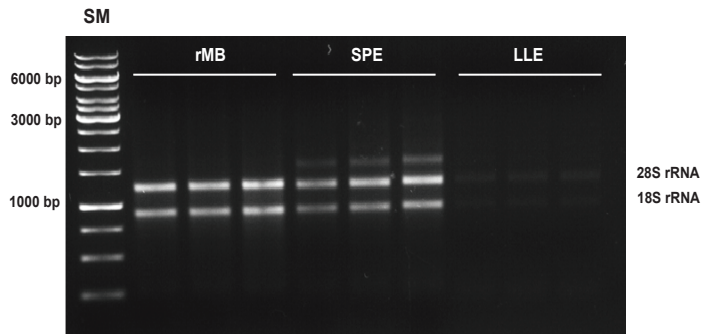

Supplement: Supplementary file 5 — Additional file 5: Figure S2. Agarose gel electrophoresis for RNA samples extracted using either the robot-automated magnetic binding (rMB), solid phase extraction (SPE) or liquid–liquid extraction (LLE) methods. The 28S and 18S rRNA bands are indicated in the right border, and the size marker (SM) is presented in the first lane. An example of one representative biological repeat out of three performed is presented. [file 13071_2022_5624_MOESM5_ESM.pdf]

Trophozoites

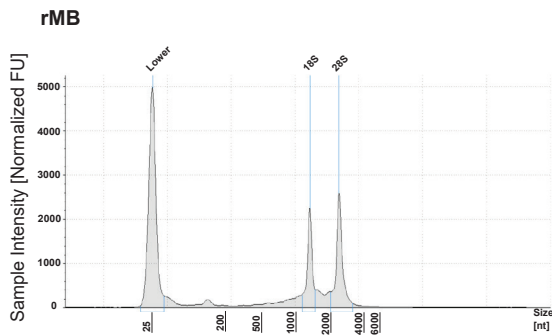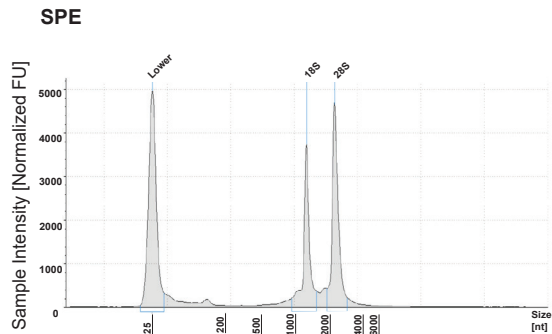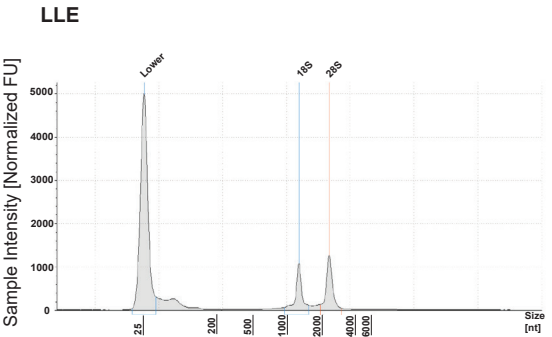

Rings

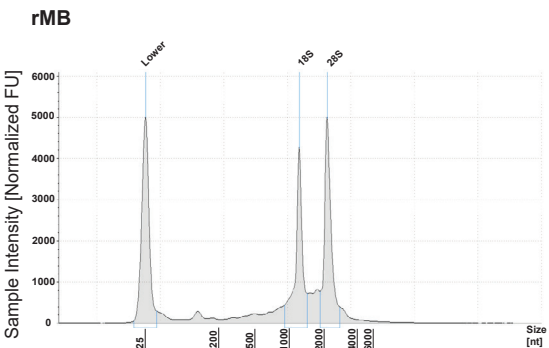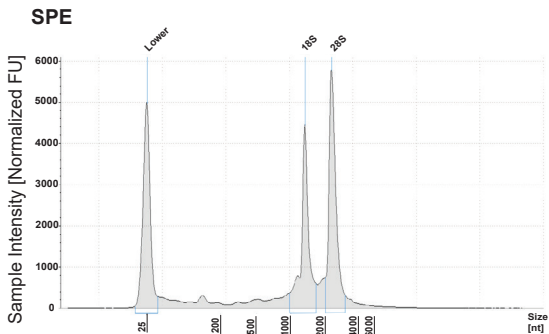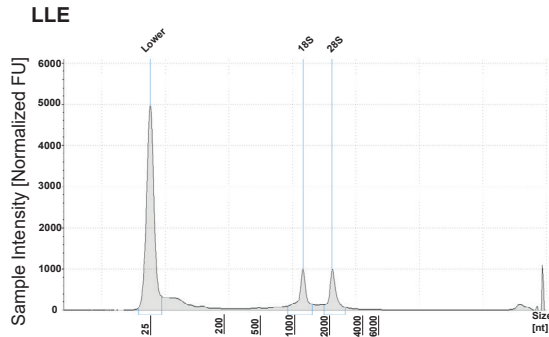

Supplement: Supplementary file 6 — Additional file 6: Figure S3. Capillary electropherograms for one representative sample of each biological replicate. Importantly, all of the LLE samples presented RNA concentrations much lower than the rMB and SPE methods in the quantification provided by capillary electrophoresis (data not shown). This suggests that the RNA quantification based on spectrophotometry at 260 nm (Fig. 1a, b) for the LLE samples largely overestimates its actual concentration, probably because of a large degree of protein contamination. Proteins also absorb at 260 nm and can interfere with RNA quantification when present in large quantities [68, 88] as revealed in the A260/A280 purity ratio for this method. [file 13071_2022_5624_MOESM6_ESM.pdf]

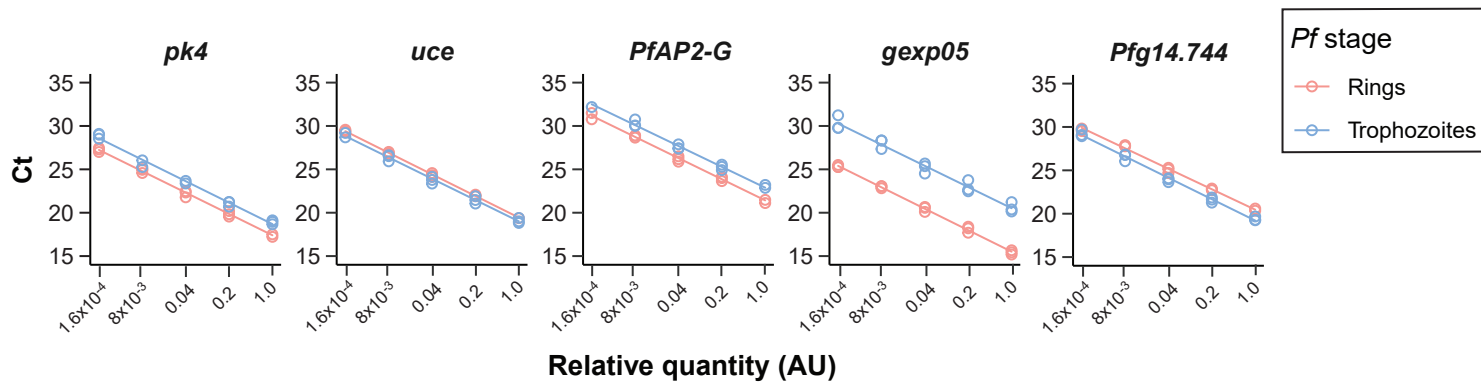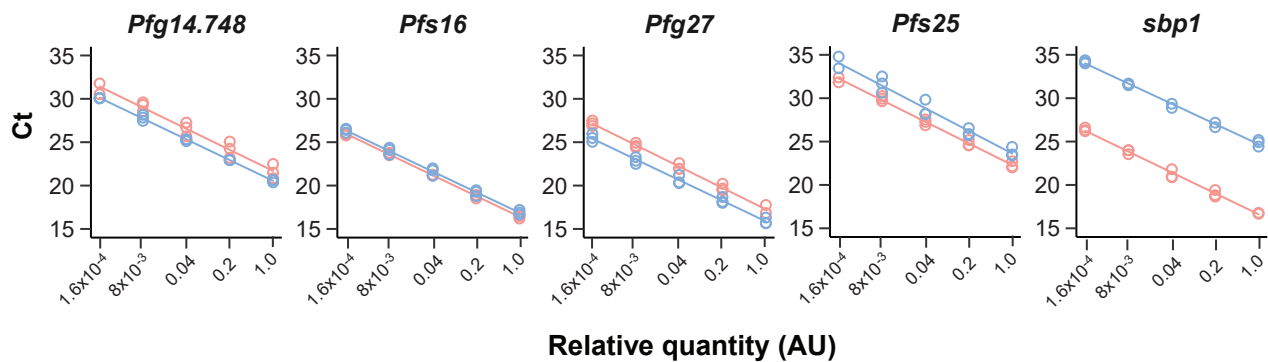

Supplement: Supplementary file 7 — Additional file 7: Figure S4. Mean standard curves for the GRG panel in ring and trophozoite stage parasites in three independent biological repeats (n = 3). Standard curves were entirely generated by means of the robotic system, by a serial 1:5 dilution of a standard pool of ring- or trophozoite-derived cDNA. The statistical parameters of one representative example of each biological repeat, used for actual relative quantification, are presented in Additional file 2: Table S2. The statistical correlation and efficiency parameters of the average of the three repeats in a mixed model ANOVA for batch effect were used to evaluate reproducibility of the standard curves, and are presented in Additional file 3: Table S3. Data represent the mean of three biological replicates (n = 3) with three technical replicates each. Error bars represent SD. [file 13071_2022_5624_MOESM7_ESM.pdf]

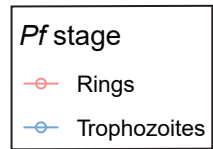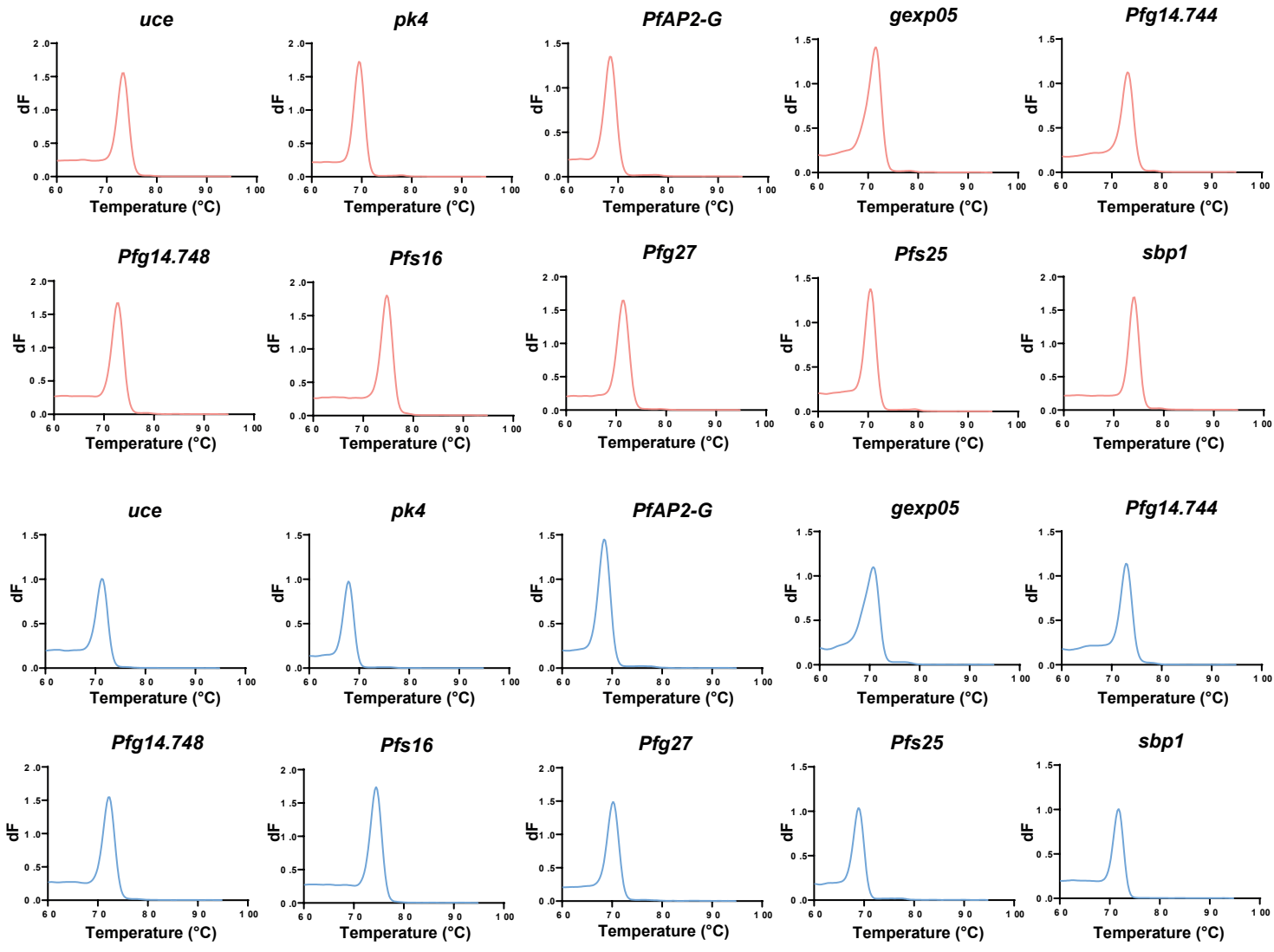

Supplement: Supplementary file 8 — Additional file 8: Figure S5. Representative melting curves of the early gametocytogenesis gene panel applied to ring and trophozoite stage parasites. The derivative of the reporter dye fluorescence (dF) against temperature is presented. [file 13071_2022_5624_MOESM8_ESM.pdf]

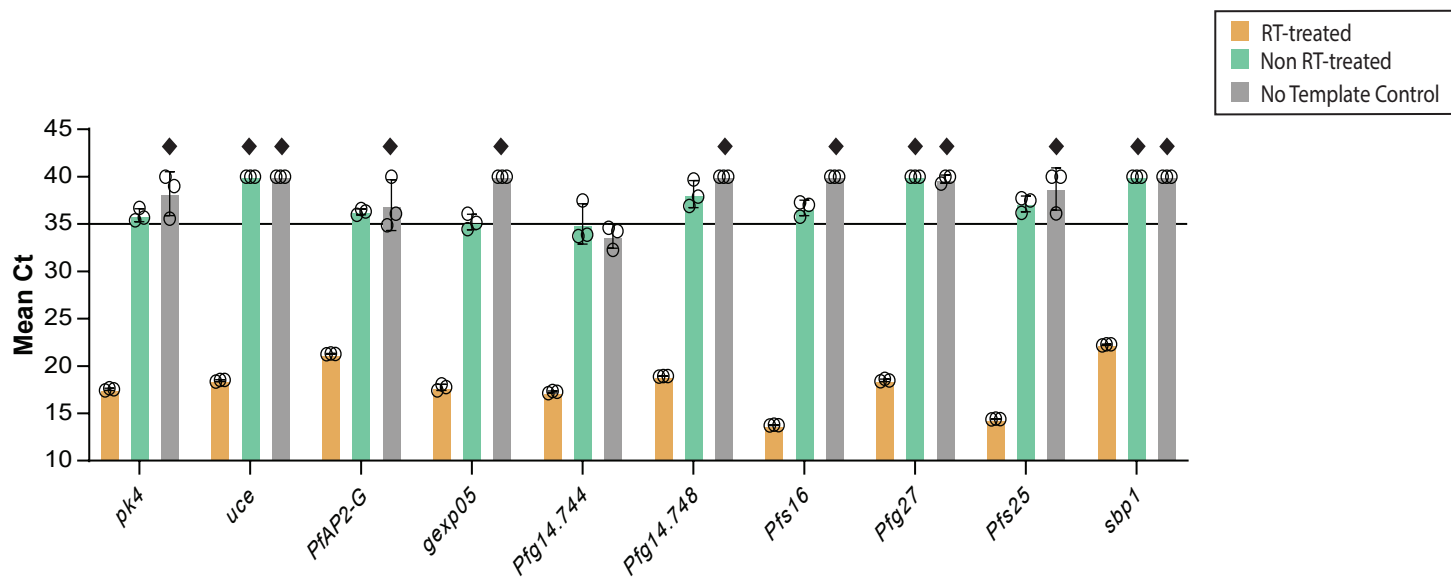

Supplement: Supplementary file 9 — Additional file 9: Figure S6. No reverse transcription RT-qPCR assay. RNA extracted from trophozoite-stage parasites was subjected to reverse transcription to generate cDNA (RT-treated) or directly used as input (Non RT-treated) for the RT-qPCR reaction, using a no-template control as a control for no amplification. Amplification was considered negligible if the Ct value was above the low amplification threshold of Ct = 35 [63] (represented as a horizontal line in the plot). For those repeats where amplification was negative (Ct ≥ 40), the Ct value was considered equal to 40.0. The symbol ♦ represents those bars where at least one of the biological repeats did not present any amplification and was thus considered Ct = 40. Data represent the mean of three biological replicates (n = 3) with three technical replicates each. Error bars represent SD. [file 13071_2022_5624_MOESM9_ESM.pdf]

**a**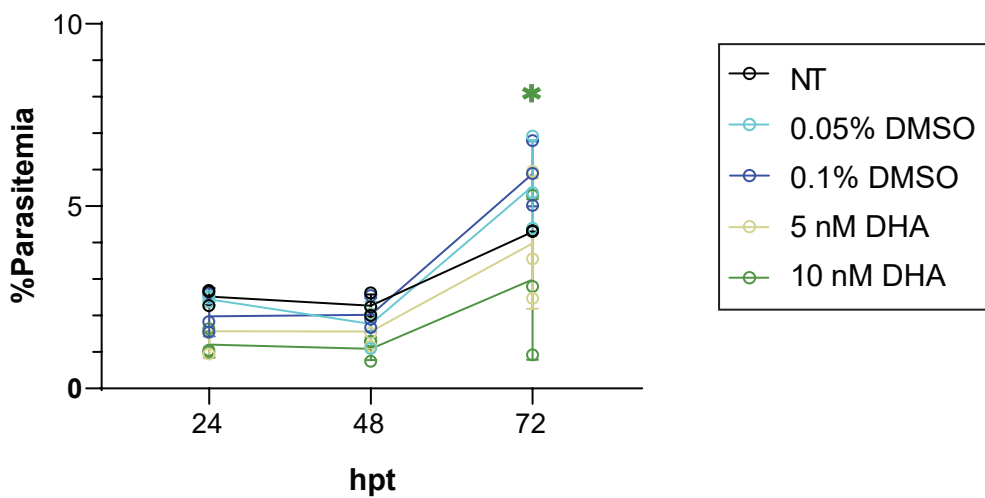**b**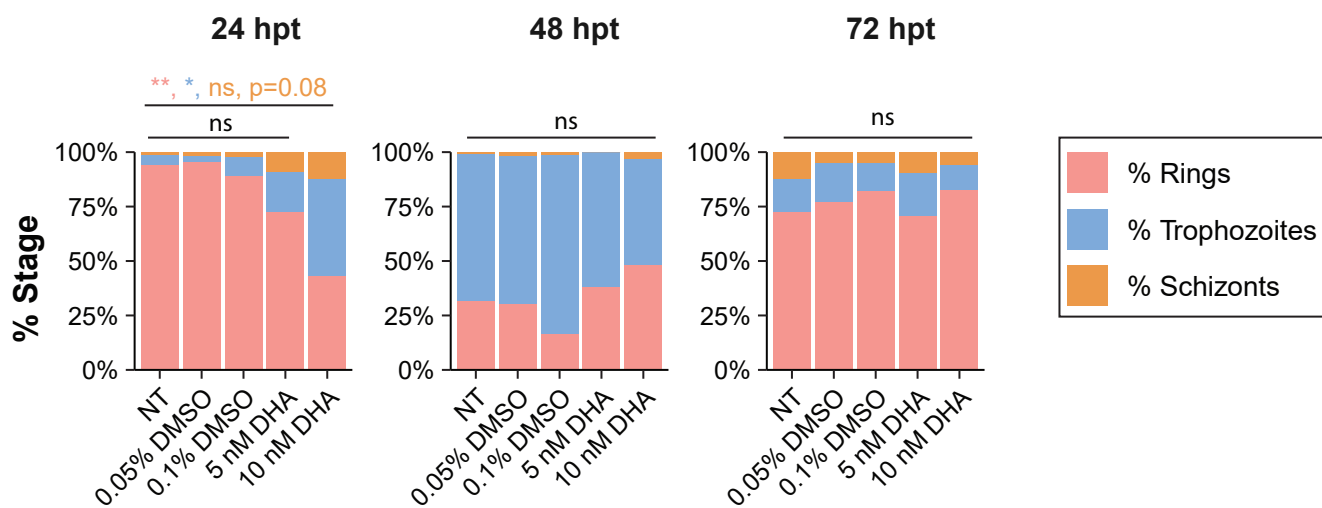

Supplement: Supplementary file 10 — Additional file 10: Figure S7. Growth dynamics and stage composition analysis during DHA treatment. a Parasitemia quantified by Giemsa-stained smears for the growth assay presented in Fig. 3H. Parasitemia was determined by manual counting of Pf-iRBC percentage relative to the total RBC in least 10 fields, in three technical replicates of each one of the three independent biological replicates. b Stage composition analysis of the Giemsa-stained smears of the growth assay after 24, 48 or 72 hpt. Mean percentages of ring, trophozoite and schizont stage parasites were calculated in at least 50 parasites over 10 fields in three technical replicates of each one of the three independent biological replicates. Error bars represent SD. Two-way ANOVA with post-hoc tests were run using estimated marginal means with the R package ‘emmeans’ for Panel a, and one-way ANOVA with Dunnett’s multiple comparisons test was performed for each stage in each time point independently in Panel b. * = p < 0.05 and ** = p ≤ 0.01. [file 13071_2022_5624_MOESM10_ESM.pdf]

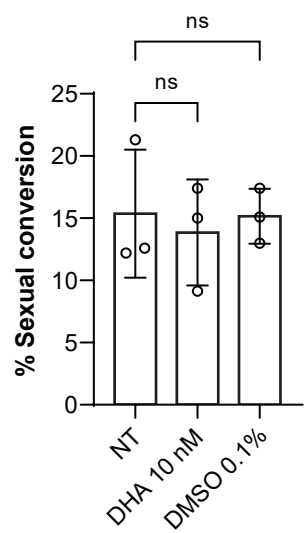

Supplement: Supplementary file 11 — Additional file 11: Figure S8. Sexual assay to measure Pf sexual conversion. NF54 trophozoite-stage parasites were treated with DHA 10 nM, DMSO 0.1% (solvent control) or non-treated. Media were replaced daily for 7 days supplemented with 10% human serum and NAG. Asexual % parasitemia and % gametocytemia were determined manually on day 1 and day 7, respectively, using Giemsa-stained blood smear counting over 10 fields. Percentage of sexual conversion was calculated by dividing the day 7% gametocytemia by the day 1% asexual parasitemia and multiplying by a factor of 100%. Data represent the mean of three independent biological repeats, the error bars represent SD, and one-way ANOVA with Dunnett’s multiple comparisons test was performed. ns = p > 0.05. [file 13071_2022_5624_MOESM11_ESM.pdf]

**a**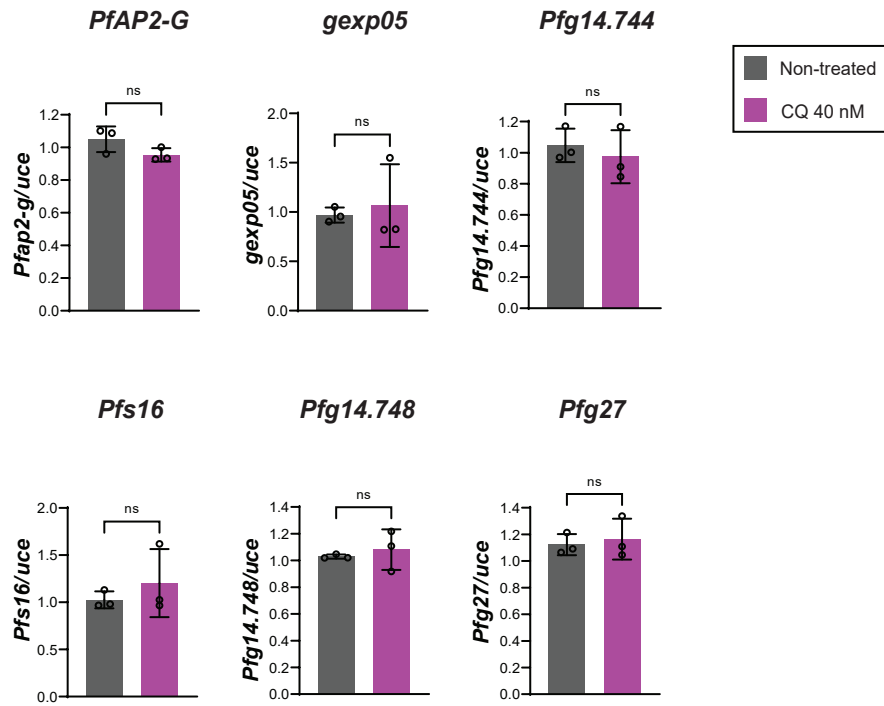**b**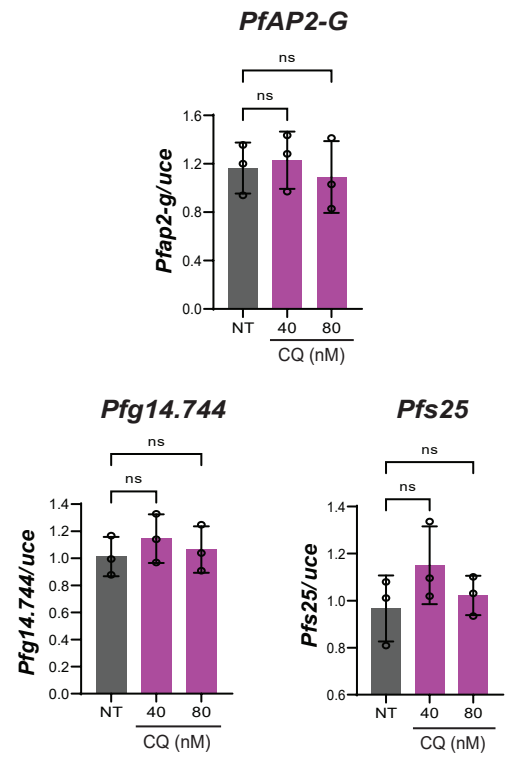

Supplement: Supplementary file 12 — Additional file 12: Figure S9. Early gametocytogenesis gene expression screening with chloroquine. a Mean gene expression of PfAP2-G, gexp05, Pfg14.744 Pfg14.748, Pfs16 and Pfs25 of ring-stage Pf cultures exposed to 40 nM choloroquine (CQ) for 24 h or non-treated (NT); data were normalized to uce. b Mean gene expression of the parasitic genes PfAP2-G, Pfg14.744 and Pfs25 of trophozoite-stage cultures exposed to increasing concentrations of CQ over the course of 4 h or non-treated (NT); data were normalized to uce. Data represent the mean of three independent biological repeats. Error bars represent SD. One-way ANOVA with Dunnett’s multiple comparisons test was performed for Panel b, and an unpaired t-test was performed for Panel a. P-values were adjusted for batch effect in Panel a. ns represents p ≥ 0.05 (non-significant). [file 13071_2022_5624_MOESM12_ESM.pdf]

**a**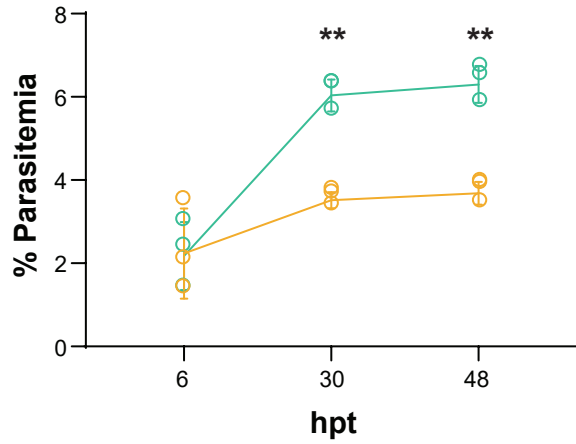**b**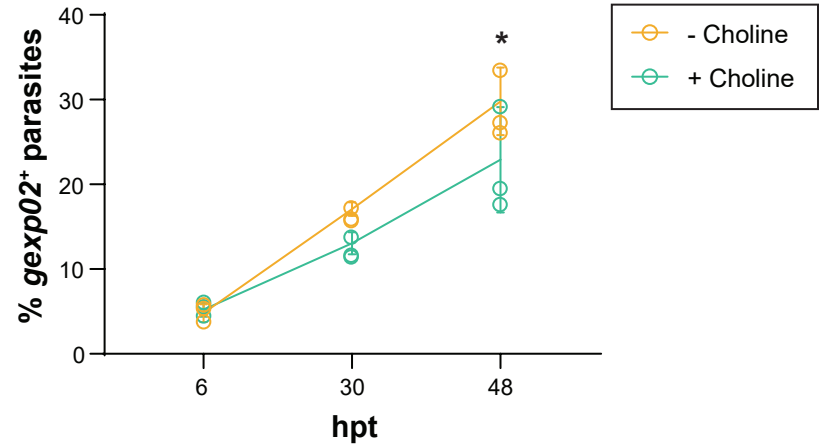**c**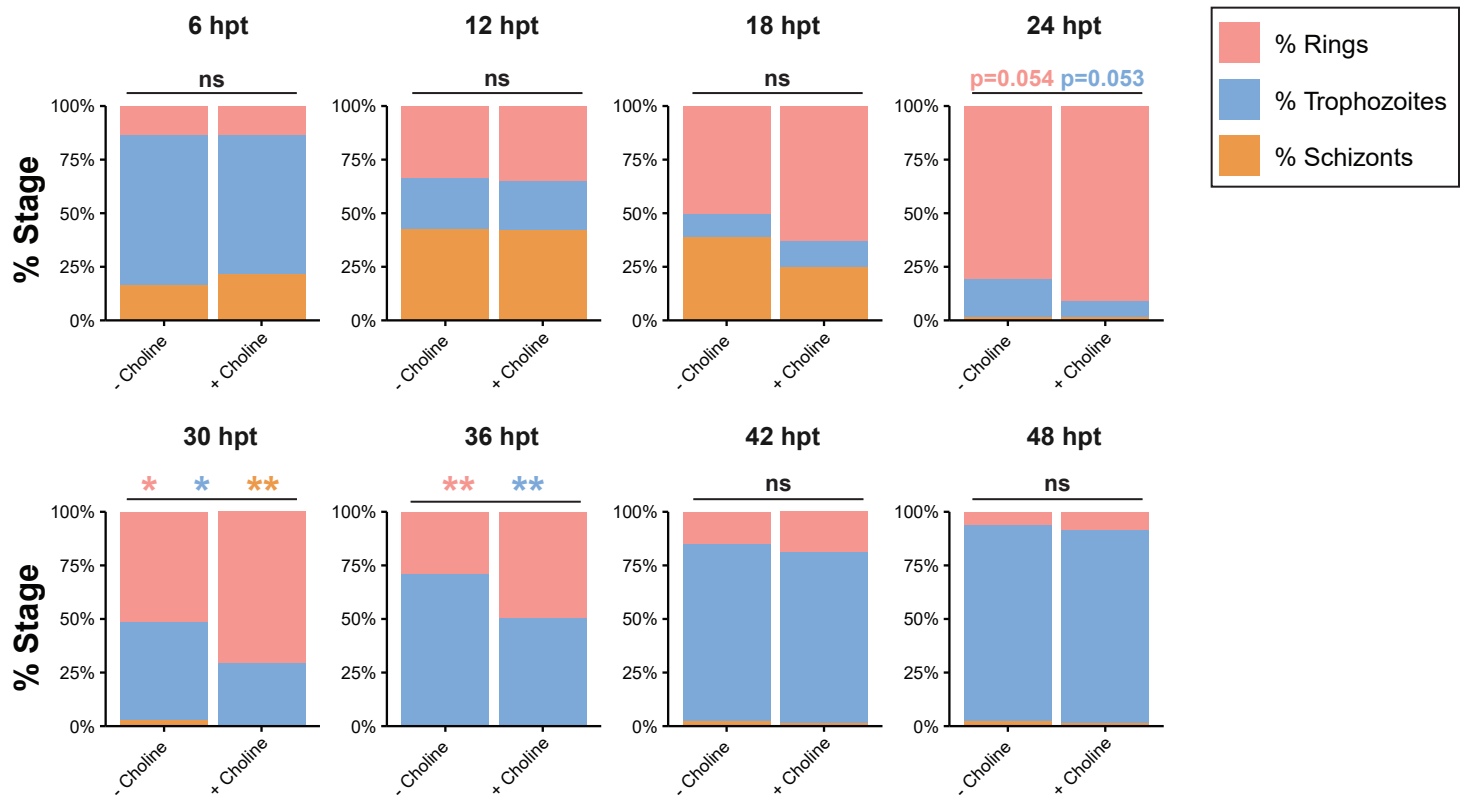**d**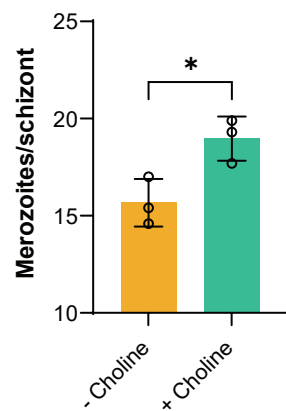**e**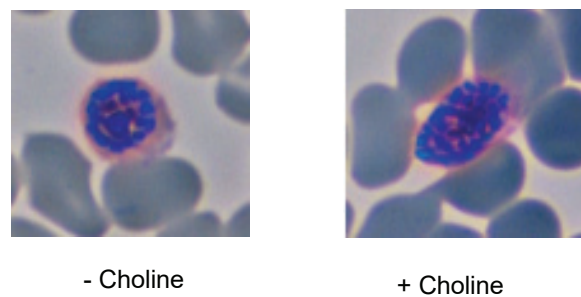

Supplement: Supplementary file 13 — Additional file 13: Figure S10. Growth dynamics and stage composition analysis during choline treatment. a Parasitemia and b Percentage of gexp02+ iRBC quantified by flow cytometry for the experiment presented in Fig. 4. Parasitemia was determined by the percentage of gated Hoechst-33342 positive Pf-infected cells and %gexp02+ as the percentage of gated Hoechst-33342 and tdTomato+ double-positive cells, as explained in the Methods section. c Stage composition analysis of the Giemsa-stained smears obtained in each one of the time points evaluated, as illustrated in Fig. 4. Mean percentages of ring, trophozoite and schizont stage parasites were calculated in at least 50 parasites over 10 fields in three technical replicates of each one of the three independent biological replicates. d Mean number of merozoite daughter cells per schizont, quantified at 12 hpt in the choline-depleted and -supplemented cultures (Fig. 4). Mean merozoites/schizonts were quantified in at least 10 representative schizonts in each one of the three biological replicates per treatment. e Representative microscopic images of schizonts of choline-depleted and -supplemented cultures in Giemsa-stained smears. Error bars represent SD. Two-way ANOVA with post-hoc tests were run using estimated marginal means with the R package ‘emmeans’ (Panels a and b). A one-way ANOVA with Dunnett’s multiple comparisons test was performed for each stage in each time point independently (Panel a), and an unpaired ttest was performed (Panel d).*p < 0.05 and **p ≤ 0.01. [file 13071_2022_5624_MOESM13_ESM.pdf]

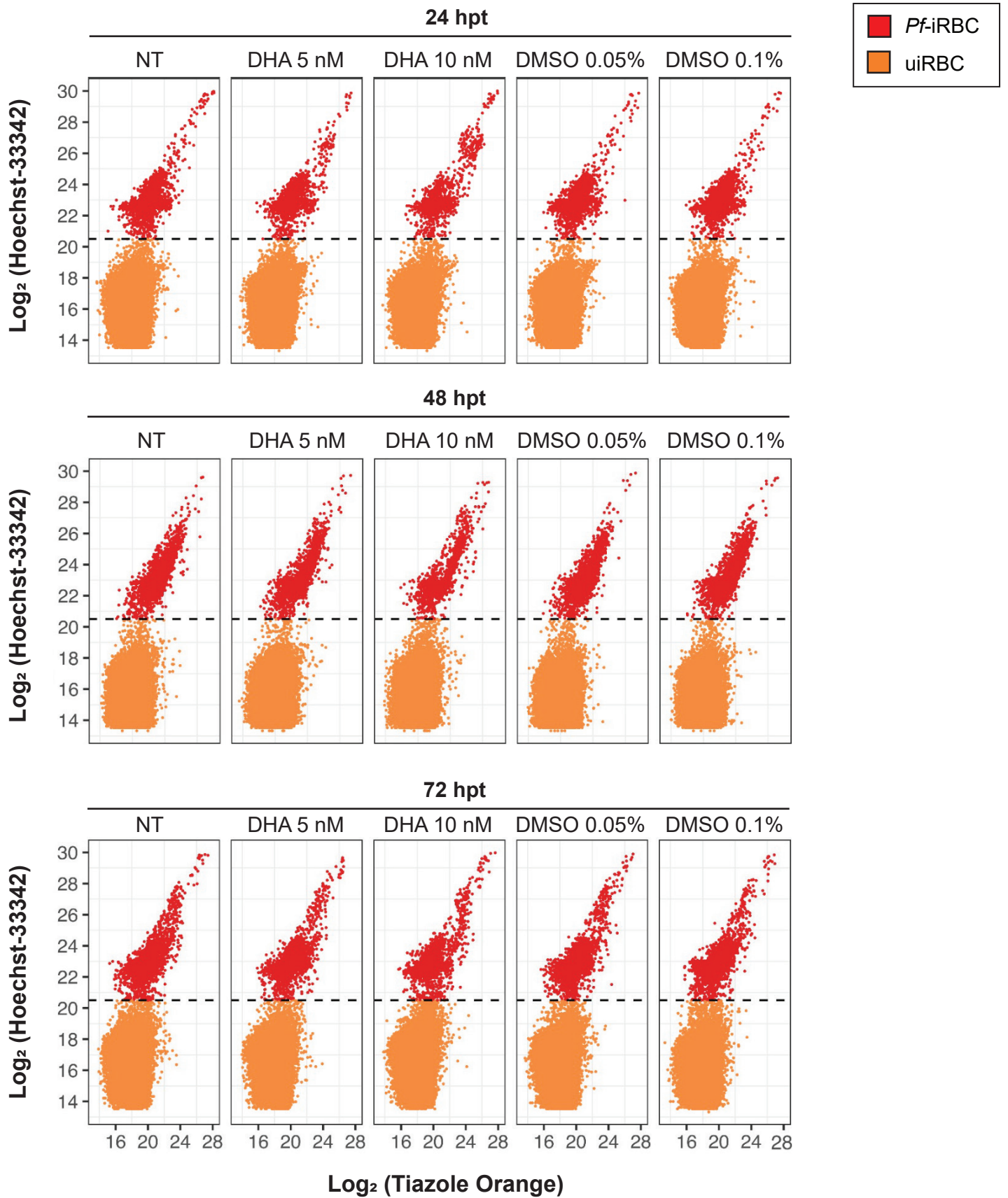

Supplement: Supplementary file 14 — Additional file 14: Figure S11. Population scatter plots and flow cytometry gating strategy for the DHA growth assay. NF54 Pf-iRBCs were treated as described in Fig. 3. Cells were harvested and stained with Hoechst-33342 and thiazole orange. Initially, a viable singlet RBC population (R1) was gated in the forward scatter vs. side scatter plot (not shown) to exclude doublets and cell debris. The singlet infected RBC population (R2) gating strategy is presented in this figure, with the thiazole orange (TO) log2 fluorescence intensity in the x-axis and the Hoechst-33342 log2 fluorescence intensity in the y-axis of each scatter plot. The population of Pf-iRBCs was considered to be those cells positive for the DNA stain Hoechst 33342 (gated in red) and the RNA stain. TO staining served as a control for non-specific staining (TO+ only, non-gated). Parasitemia was calculated as the percentage of Pf-iRBCs from the total initial singlet RBC population (uninfected RBCs). Analysis was performed using a ZE5™ flow cytometer (Bio-Rad). This figure presents representative scatter plots from one technical repeat out of three performed per biological repeat. [file 13071_2022_5624_MOESM14_ESM.pdf]
